# Quantifying the financial burden of heat-related hospital admissions in Switzerland under a changing climate: A scalable analytical framework

**DOI:** 10.1186/s44263-026-00275-w

**Published:** 2026-05-28

**Authors:** Ario Saeid Vaghefi, Iris Bucher, Chiara Colesanti Senni, Veruska Muccione, Martina S. Ragettli, Abbas Mirmashhouri, Christian Huggel, Markus Leippold

**Affiliations:** 1 Department of Finance, University of Zürich, Plattenstrasse 14, 8032 Zürich, Switzerland; 2 Department of Geography, University of Zürich, Winterthurerstrasse 190, 8057 Zürich, Switzerland; 3 SCOR, Reinsurance, Claridenstrasse 4, 8002 Zürich, Switzerland; 4 Swiss Federal Institute for Forest, Snow and Landscape Research (WSL), Züricherstrasse 111, 8903 Birmensdorf, Switzerland; 5 Swiss Tropical and Public Health Institute (Swiss TPH), Kreuzstrasse 2, 4123 Allschwil, Switzerland; 6 Department of Epidemiology and Public Health, University of Basel, Totengässlein 3, 4051 Basel, Switzerland; 7 Swiss Finance Institute (SFI), Walchestrasse 9, 8006 Zürich, Switzerland

**Keywords:** Heat-related morbidity, Climate change health costs, Distributed Lag Non-Linear Model (DLNM), SwissDRG / healthcare cost projection

## Abstract

**Background:**

While heat-related mortality is well-documented, the full economic burden of non-fatal illness remains underexplored. This gap hinders evidence-based health planning in a warming climate. We aimed to quantify the current and projected financial burden of heat-related hospital admissions in Switzerland.

**Methods:**

We linked daily hospital admissions (1998–2022) from six Swiss cantons, representing 60% of the national total, to temperature records using a Distributed Lag Non-Linear Model (DLNM) meta-analysis. Costs were estimated via the Swiss Diagnosis-Related Groups (SwissDRG) tariff system across different disease categories and age groups. Future climate projections were generated using CH2018 climate simulations with Shared Socioeconomic Pathway (SSP) scenarios (SSP1–Representative Concentration Pathway (RCP)2.6, SSP2–RCP4.5, SSP5–RCP8.5).

**Results:**

Historically, extreme heat significantly increased hospital admissions in major Swiss regions, with endocrine/metabolic disorders showing the highest relative risk (RR 2.02). The elderly (75+) and children were the most vulnerable populations. The average annual cost of these direct hospitalizations across the six cantons studied was Swiss Francs (CHF) 20.6 million (2013–2022), notably a conservative estimate representing only a fraction of the total economic burden. Future projections show this burden escalating sharply. Under a high-emissions pathway (SSP5-RCP8.5), these direct costs are projected to increase 2.5-fold by the 2060s, with costs for the elderly quintupling. Critically, even with aggressive mitigation (SSP1-RCP2.6), costs are still projected to triple compared to the baseline. This is driven primarily by demographic aging, with climate change acting as a significant amplifier, responsible for 15–30% of the projected cost increases for the elderly.

**Conclusions:**

Our findings from major Swiss regions reveal a substantial and growing financial burden on the healthcare system. Given that these figures represent a *lower bound*, the true costs are likely much higher. This evidence underscores the urgent need for nationally coordinated adaptation policies to protect public health and ensure healthcare sustainability, even as mitigation efforts continue.

## Background

Climate change is increasingly recognized as a major threat to public health, with rising temperatures and more frequent, intense heatwaves placing significant strain on healthcare systems worldwide [1–4]. According to the World Meteorological Organization (WMO), extreme heat is now the deadliest form of extreme weather, underlining the urgency of integrating climate services into public health planning [3]. The World Health Organization (WHO) projects that climate change will lead to approximately 250,000 additional deaths annually between 2030 and 2050 due to heat stress, malnutrition, and related impacts [5]. Recent projections suggest that if current warming trajectories continue, over half of the population born in 2020 will experience unprecedented lifetime exposure to extreme heat events, with potentially severe implications for global health equity [6].

Even in high-income countries like Switzerland with a per capita gross domestic product (GDP) of approximately United States Dollars (USD) 105,000 in 2025 [7], a globally strong insurance and banking sector accounting for approximately 9% of GDP, and a life-sciences industry contributing nearly 6% of GDP and employing over 135,000 people [8], extreme heat events are exerting growing pressure on economic and health infrastructure. Despite its comprehensive and costly healthcare system (around 12% of GDP, Swiss Francs (CHF) 99 billion in annual spending) and mandatory insurance coverage [9], Switzerland continues to face significant heat-related burdens. While the impact of heat on mortality, often highlighted during individual extreme summers, has been well documented [10–14], its effects on morbidity, particularly hospital admissions and associated healthcare costs, remain less explored. Switzerland, with one of the highest per-capita GDPs globally and a strong insurance and pharmaceutical industry, might be expected to be well-protected. However, recent analyses reveal that even today’s climate causes substantial economic strain: heat-related reductions in labour productivity cost around CHF 665 million annually [15], while extreme heat events are projected to add an estimated USD 1.1 trillion to global health-system costs by 2050, including those in developed nations like Switzerland [16]. The Swiss Federal Office for Environment (BAFU) identifies health impacts, especially heat-related mortality, as the most severe climate-related risk for the country [17], and heat-related mortality is now monitored through a national climate impact indicator [18]. Understanding these domestic impacts is crucial for developing effective public health strategies and adaptation measures.

Globally, studies have established links between observed heat exposure and increased hospital admissions for a variety of health conditions, including metabolic disorders, renal failure, and sepsis, often exacerbated by humidity and pollution [19, 20]. In the United States, for instance, extreme heat has been linked to elevated emergency visits for renal and mental health issues [21–23]. However, the impact on cardiovascular and respiratory admissions shows more varied results, with some studies indicating no consistent increases or even decreases during hot days [24, 25], while broader meta-analyses confirm a positive association between temperature and cardiovascular morbidity, especially among older adults [26]. These findings provide strong evidence that current and past heat exposure is already impacting hospital systems across multiple health dimensions, underscoring the urgent need to anticipate and quantify how such impacts may evolve in the future. Yet, while heat-related hospital admissions have been widely documented, forward-looking projections of such admissions, especially under varying climate and demographic scenarios, remain scarce. We use the term relative risk (RR) throughout this paper to denote the cumulative hospitalization risk at a given temperature relative to a reference temperature.

Projections for future heat-related mortality suggest significant increases, particularly when demographic changes like population aging are considered [11, 12, 27]. However, comprehensive projections of future hospitalizations (morbidity) and their associated costs are less common. Existing economic analyses have often focused on historical burdens for specific conditions or regions [28–31], with few studies providing detailed future cost projections under various climate and demographic scenarios [32–34]. Nonetheless, important exceptions exist: the Lancet Countdown reports [35, 36] have highlighted anticipated economic and healthcare burdens due to rising heat exposure, though with limited disease-specific hospitalization estimates. Similarly, Limaye et al. [37] provided a detailed analysis of the health-related economic costs of climate-sensitive events, including morbidity, offering a methodological foundation for future projections. These studies provide important context and illustrate the growing recognition of heat as a systemic threat to health systems globally.

Switzerland, despite its temperate climate, has experienced significant warming over recent decades—with near-surface temperatures rising by around 2.9^∘^C since preindustrial times, summer heatwaves in low-lying and urban areas now occurring with much greater frequency and intensity, and all nine warmest years on record observed post-2010 [38, 39]. Despite the well-resourced healthcare system, the 2003 European heatwave resulted in hundreds of excess deaths in Switzerland, and in 2023, over 500 heat-related fatalities were reported [14, 40]. Previous Swiss studies have found increased emergency hospital admissions (EHAs) during heatwaves for conditions like renal and infectious diseases, and more broadly, heat has been attributed to 1.1% of all EHAs, with dehydration and acute kidney injury being particularly affected [41, 42]. However, a comprehensive, long-term quantification of heat-attributable hospitalizations across all major disease categories, their evolution over time and under different climate scenarios, and the associated financial implications for the Swiss healthcare system remains largely unexamined. This gap leaves policymakers without crucial data for robust adaptation planning and resource allocation.

This study aims to fill this knowledge gap by quantifying the impact of heat on hospital admissions in Switzerland over a 25-year period (1998–2022), assessing how this burden has changed over time, and estimating the associated healthcare costs. Using nationwide daily hospital admission data and meteorological records, we apply a two-stage statistical approach involving Distributed Lag Non-Linear Models (DLNMs) [43] and multivariate meta-regression to quantify the RR of hospitalization at different temperature exposures.

## Methods

### Study design

We conducted a nationwide time-series analysis to quantify the association between ambient temperature and hospital admissions across Switzerland from 1998 to 2022. This study was designed to evaluate both the epidemiological burden of heat-related morbidity and its economic implications for the Swiss healthcare system.

Our analysis is grounded in the following conceptual causal pathway linking climate change to healthcare costs: greenhouse gas emissions drive temperature rise, leading to increased heat exposure (measured as daily maximum temperature, *T*_*max*_). Elevated temperatures induce physiological stress, which may precipitate clinical events resulting in hospital admission. These admissions generate economic costs, quantified via the SwissDRG tariff system. Our statistical framework focuses on estimating the exposure-response relationship between temperature and hospitalization, with costs derived subsequently from observed admission patterns.

To estimate the temperature-hospitalization relationship, we employed a widely adopted two-stage analytical framework. In the first stage, we applied DLNMs at the cantonal level, stratified by disease groups and age categories. DLNMs are a flexible modeling approach commonly used in environmental epidemiology to characterize non-linear and delayed effects of environmental exposures on health outcomes [43, 44]. This method captures both the shape of the temperature–response function and the distributed effects over a lag period, reflecting the delayed physiological impacts of heat exposure. Models were adjusted for long-term trends, seasonality, day of the week, and national holidays to reduce confounding.

In the second stage, we pooled the canton-specific exposure-response associations using multivariate meta-regression. This approach enables the derivation of national and age-specific risk estimates while accounting for between-canton heterogeneity and borrowing statistical strength across regions [44].

To quantify the financial burden of heat-related hospitalizations, we linked the epidemiological findings with economic data. Hospital admission records, which include SwissDRG codes from 2012 onwards, were used to assign costs to each hospitalization. The SwissDRG system standardizes reimbursement for inpatient acute-care hospital services by assigning cost weights to diagnostic groups (DRGs), which reflect the relative resource use of different hospital cases. These weights are then multiplied by canton-specific base rates to produce standardized estimates of treatment costs. This approach allowed us to calculate the proportion of healthcare costs attributable to heat exposure (see the Statistical Analysis subsection of the Methods for details on cost estimation).

Furthermore, to anticipate future challenges, we incorporate high-resolution climate projections from the CH2018 climate scenarios [45, 46] and Shared Socioeconomic Pathways (SSPs) to estimate future hospitalization rates and associated healthcare costs under different climate change scenarios.

Lastly, we projected future heat-related hospitalizations and associated costs under different climate and socioeconomic pathways. Projections were based on high-resolution temperature simulations from the CH2018 climate scenarios for Switzerland and incorporated demographic changes using SSP population projections. This forward-looking component offers a basis for anticipating future healthcare burdens under evolving climate and demographic conditions.

### Data

#### Hospitalization data

Hospital admission records were obtained from the Swiss Federal Office of Statistics. Data use was approved under contract No. 240448 with the Swiss Federal Statistical Office (BFS); further details are provided in the Declarations section. The dataset includes all-cause and cause-specific hospitalizations from 1998 to 2022, with diagnoses coded according to the International Classification of Diseases, 10th Revision (ICD-10) [47]. For the period 2012–2022, each admission is also assigned a Swiss DRG code, which reflects the cost of treatment under the national tariff system. Demographic information includes patient age (in five-year intervals) and gender. We focused on hospitalizations from six cantons: Bern (BE), Basel (BS), Geneva (GE), Ticino (TI), Vaud (VD), and Zürich (ZH), which encompass the most densely populated urban regions in Switzerland. Together, these cantons account for approximately 60% of all hospital admissions recorded nationwide during the 1998–2022 period, and have also been the focus of previous clinical research on heat-related cardiovascular morbidity and mortality in Switzerland [24].

#### Meteorological data

Daily meteorological data, including maximum and minimum temperature (${T}_{\max },{T}_{\min }$) measurements, were obtained from MeteoSwiss, Switzerland’s national meteorological service. Historical temperature data were derived from quality-controlled observations at nationally recognized meteorological stations. Future climate conditions were based on temperature projections from the CH2018 dataset [45, 46], which provides downscaled and bias-adjusted climate scenarios for Switzerland (2020–2100). For each canton, one station considered to be broadly representative of regional climate conditions was selected, following the approach used in the CH2018 cantonal factsheets. These stations, while not totally optimized for heat stress analysis, provide a standardized basis for inter-cantonal comparison. The list of selected stations is available via the National Centre for Climate Services (NCCS) cantonal factsheets (https://www.nccs.admin.ch/nccs/en/home/regions/kantone.html).

#### Cost data: SwissDRG

The economic impact of heat-related hospitalizations was estimated using SwissDRG tariff data. This analysis adopts a payer-based perspective, utilizing tariffs that reflect the direct reimbursement burden on the healthcare system and insurers, rather than a full societal cost-of-illness approach. The data is publicly available on the SwissDRG website (www.swissdrg.org) and updated yearly. The SwissDRG system provides reimbursement rates for inpatient acute-care hospital services based on Diagnosis Related Groups (DRGs). Each DRG is assigned a cost weight, which is updated annually based on hospital cost and service data. The total hospitalization cost for each case is computed as:

1 $${\rm{Cost}}=\,\text{cost weight}\times \text{base rate}\,,$$

where the base rate varies by canton and is approved by local authorities. We calculated a cantonal-level cost estimate by averaging the base rates across all hospitals within each canton. The approved base rates for each hospital in the considered cantons from 2012 to 2025 are provided in Supplementary Material 1: Table S1. The data was collected from the published base rates from the official websites of each Swiss canton.

#### Sample construction

The final dataset comprises all hospital admissions from 1998 to 2022, categorized by disease group based on primary diagnosis (ICD-10), age group, and canton. We grouped the patients into the following 21 mutually exclusive disease groups: infectious and parasitic diseases (A00–B99), neoplasms (C00–D49), blood and immune system (D50–D89), diabetes (E10–E14), endocrine and metabolic diseases (E00–E10, E15–E99), Alzheimer’s and dementia (F00–F03, G30–G31.1, G31.8–G31.9), mental disorders (F04–F99), nervous system (G00–G99), eye diseases (H00–H59), ear diseases (H60–H95), circulatory system (I00–I99), chronic obstructive pulmonary disease (COPD) (J41–J44), respiratory system without COPD (J00–J40, J45–J99), digestive system (K00–K99), skin and subcutaneous tissue (L00–L99), musculoskeletal system (M00–M99), urinary system (N00–N39), reproductive organs (N40–N98), pregnancy and childbirth (O00–O99), newborns (P00–P96, Q00–Q45), and injuries, poisonings and external causes of hospitalization (S00–S99, T08–T19, T33–T99, V01–V99, X01–X99, Y01–Y99). This grouping closely follows the main ICD-10 categories, including, in addition, separated groups for diabetes, Alzheimer’s and dementia, and COPD as they have been found in previous studies to be particularly vulnerable to heat [10]. The corresponding mapping and a more detailed description are given in Supplementary Material 1: Table S2.

We aggregated the five-year age intervals into three main age groups: children and adolescents (0–14 years), adults (15–74 years), and older adults (75+ years). To ensure meaningful results, we excluded disease-age combinations with less than 100 observations per canton throughout the whole period: Alzheimer’s and dementia in the 0–14 age group, COPD in the 0–14 age group, pregnancy and childbirth in the 0–14 and 75+ age groups, and newborns in the 15–74 and 75+ age groups.

### Statistical analysis

#### Temperature-morbidity relationship: Two-stage time-series meta-analysis

We estimated the relationship between temperature and morbidity using a two-stage time-series approach. In the first stage, we applied a DLNM with quasi-Poisson regression to quantify the non-linear and delayed effects of daily maximum temperature (${T}_{\max }$) on hospital admissions [43]. The DLNM framework flexibly models both the exposure-response relationship and the lag structure simultaneously through a bi-dimensional cross-basis function, which is critical for assessing heat-related health risks that may accumulate over several days [44]. We used year-round data to estimate the full temperature–morbidity association and subsequently restricted the analysis to heat-related effects by focusing on days above the national warm-season (May–September) median maximum temperature. Temperature was modeled using a quadratic B-spline with knots placed at the minimum, median, and maximum of the year-round temperature distribution across cantons. The lag-response curve was modeled using a natural cubic spline with two internal knots on the log scale, with a 7-day lag window based on previous literature [42, 48]. The selection of knots and percentiles was based on quasi-likelihood Akaike and Bayesian information criteria. A sensitivity analysis was conducted to examine different temperature metrics, lags, knots, and degrees of freedom. To control for temporal confounding, we included indicator variables for day of the week, month, and national holidays. The model was:

2 $$\log \left(\mu \right)=\alpha +{\rm{cb}}({T}_{max})+{\rm{dow}}+{\rm{month}}+{\rm{holiday}}$$

where $\mu ={\mathbb{E}}[y]$ denotes expected hospital admissions, *α* the intercept, *dow* the day of the week, and cb( ⋅ ) the bi-dimensional cross-basis function of temperature *T*_*max*_ at different lags. Models were fitted separately for each combination of canton, disease group, and age group. We reduced the bi-dimensional exposure-response surface to a one-dimensional cumulative exposure-response function via the crossreduce function, centered at the same reference temperature across all cantons and disease groups. In the second stage, we pooled the first-stage estimates in a separate multivariate meta-regression model for each disease group, including 18 strata (6 cantons  × 3 age groups). We derived the best linear unbiased predictions (BLUP) of the cumulative exposure-response association [49]. The meta-predictors included the cantonal median temperature and the total hospitalization count as proxies for the population. Age groups were modeled as fixed effects and cantons as random effects, allowing estimates for cantons with limited data to shrink toward a national mean while preserving local variability. This approach yields improved, canton- and age-specific exposure-response functions, which were subsequently used for projections. Each exposure-response function was extrapolated logarithmically up to 5^∘^C beyond the historical maximum, with constant risk assumed beyond that level. Empirical confidence intervals were derived from 500 Monte Carlo simulations of estimated BLUP coefficients [50].

#### Heat relative risk and attributable fraction

The RR of hospitalization at temperature *t* was calculated as:

3 $$RR(t)=\frac{{\rm{risk}}(t)}{{\rm{risk}}({t}_{{\rm{ref}}})}$$

where *t*_ref_ is the reference temperature, defined as the median *T*_*max*_ temperature of the warm season in all cantons (23.3^∘^C in our sample). This reference serves as a representative baseline for assessing deviations in hospitalization risk due to heat exposure. By construction, *RR* = 1 corresponds to a typical warm-season day, allowing interpretation of *RR*(*t*) > 1 as excess risk relative to climatologically normal conditions. We conducted a sensitivity analysis using canton-specific medians to account for spatial adaptation. This approach differs from conventional studies using the Minimum Morbidity Temperature (MMT) as a baseline, instead focusing on deviations from typical seasonal conditions, which aligns with public health perspectives on quantifying the burdens of unusually hot days. We define heat exposure as days when the maximum daily temperature exceeds the median warm-season temperature, and then compute the average RR over these heat-exposed days. To estimate the proportion of hospitalizations attributable to heat exposure, we calculate the attributable fraction (AF):

4 $$AF=\frac{RR-1}{RR}$$

For cost analysis, heat-attributable costs are computed as:

5 $$\,\text{Heat-attributable cost}\,={\rm{cost}}\times AF$$

#### Reference period

We compare future projections to the most recent historical decade (2013–2022) to provide a realistic assessment of change, as heat-related impacts are already observable. This 10-year window offers a balance between minimizing climate variability and detecting meaningful trends [51]. Given the exceptionally warm years in recent times [13], this baseline can be considered conservative. For the cost analysis, we assume constant treatment costs over time, using 2022 values. Results were qualitatively similar when using 5- or 10-year averages, as treatment costs have remained stable under the Swiss DRG system. Heat-attributable costs during the reference period are calculated annually using actual cost and risk data, then aggregated. Empirical confidence intervals for cost projections were obtained by propagating uncertainty from the epidemiological analysis. For each combination of future climate pathway and patient group, we generated 500 Monte Carlo simulations of the projected RR and attributable fraction, assuming a multivariate normal distribution of the estimated BLUP coefficients. The resulting 95% confidence intervals thus reflect both the uncertainty in the exposure-response relationship and the variability across climate model simulations within each Shared Socioeconomic Pathways (SSP) and Representative Concentration Pathways (RCP) (SSP-RCP) scenario.

#### Climate and socioeconomic projections

We used CH2018 Switzerland’s climate projections, consisting of 68 EURO-CORDEX simulations (12, 25, and 31 simulations for RCP2.6, RCP4.5, and RCP8.5, respectively) [45]. Our analysis considers three combined socioeconomic and emissions scenarios, based on SSPs [52, 53] and RCPs [54]. The approach for jointly considering these two dimensions follows the method proposed by [55]:

SSP1-RCP2.6: Low-emissions pathway aiming to limit warming below 2^∘^C, with strong mitigation, rapid decarbonization, and renewable energy transition.

SSP2-RCP4.5: Middle-of-the-road trajectory with moderate emissions, mid-century peak followed by gradual decline due to moderate climate policies.

SSP5-RCP8.5: High-emissions pathway with minimal mitigation, continued fossil fuel reliance, and substantial warming. We include this scenario not as the most likely outcome, but as a high-end risk scenario for assessing potential impacts under limited mitigation, consistent with established practice in climate risk assessment [56, 57].

**Fig. 1:**
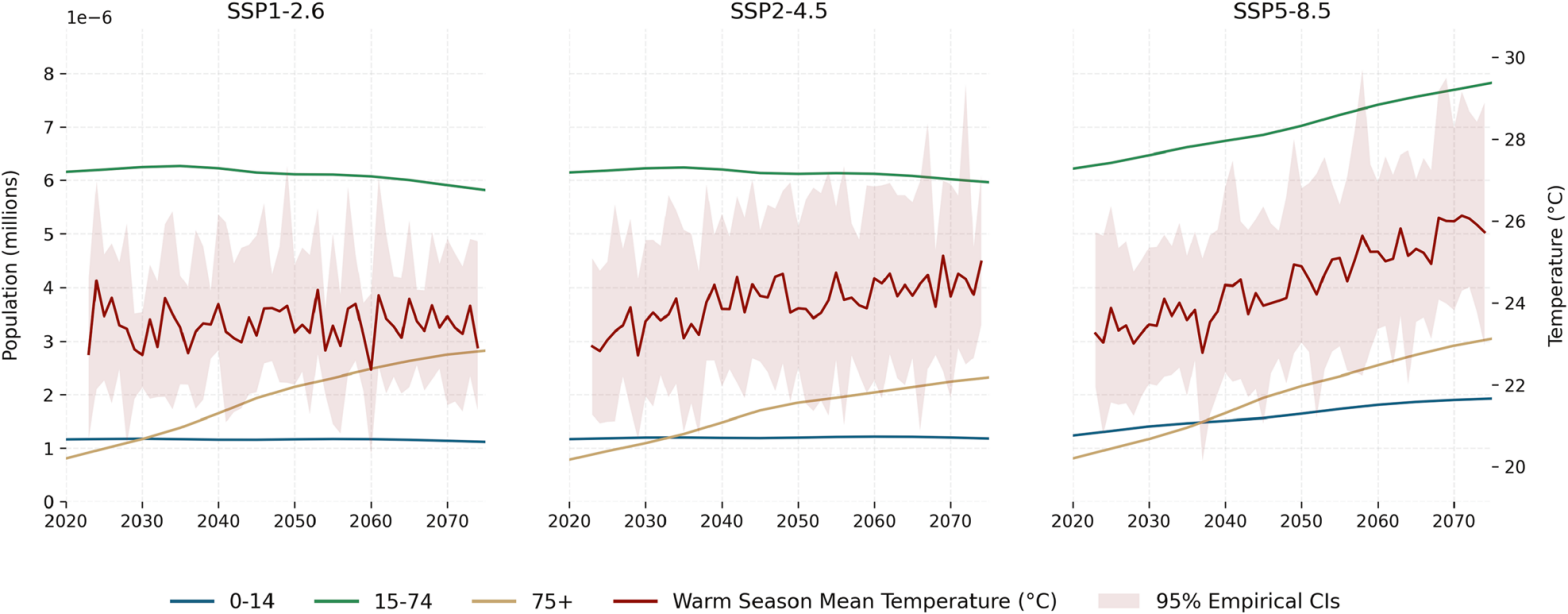
Projected annual temperature (mean *T*_*max*_ during the warm season) by RCP scenario and age-specific demographic evolution by SSP scenario

Demographic projections from the International Institute for Applied Systems Analysis (IIASA) and the National Center for Atmospheric Research (NCAR) [52] provide national-level population data by age group. We derive growth factors and apply them to each canton, assuming uniform national demographic trends. Simulations span January 2023 through December 2074. Using historical temperature-morbidity relationships, we compute RR and attributable fraction for each projected pathway. Heat-related risk is defined as average RR on days exceeding the fixed warm-season median from the historical period, considering all future days above this threshold regardless of season. Future morbidity estimates integrate demographic projections by scaling baseline hospitalization counts (historical averages during heat-exposed days) according to projected exposure days and population growth rates under corresponding SSP scenarios. Specifically, we multiply the attributable fraction by the projected number of hospitalizations to obtain the attributable number of heat-related hospitalizations under each projected temperature pathway. Figure 1 shows projected temperature and population evolution across scenarios. Under SSP1-RCP2.6, warm season mean temperature remains stable (around 23.4^∘^C). SSP2-RCP4.5 projects +1.2^∘^C increase by 2070, while SSP5-RCP8.5 expects +2.6^∘^C. Population patterns show stability or slight decline in ages 0–14 and 15–74 under SSP1-RCP2.6 and SSP2-RCP4.5, with steady growth in 75+ across all scenarios. SSP5-RCP8.5 projects sharp increases in both temperature and population across all age groups. Importantly, our projections assume constant vulnerability, meaning the exposure-response relationship estimated from historical data is held constant over time. This assumption isolates the effects of climate and demographic changes but does not account for potential future adaptation measures (e.g., improved heat warning systems, urban cooling infrastructure, behavioral changes) that could reduce heat-related health risks. As such, our projections should be interpreted as estimates of future burden under current vulnerability levels, representing an upper bound if effective adaptation is implemented.

#### Estimation of hospitalization cost

Treatment costs were computed using the SwissDRG tariff system, which standardizes reimbursement across Swiss hospitals. SwissDRG groups hospital cases with similar cost structures into DRGs, each assigned a cost-weight reflecting average resource consumption. The DRG-specific cost is calculated as:

6 $$\begin{array}{l}\,\text{Case-specific remuneration}=\text{cost weight of DRG flat-rate}\\\times \text{negotiated base rate}\,\end{array}$$

The base rate varies by hospital and is negotiated between providers and insurers, subject to cantonal approval. Further details on the SwissDRG system are available at https://www.swissdrg.org/de/akutsomatik/swissdrg. Since, in practice, the distinction between hospital costs and other service areas is not always clear, tariff partners, regulators, and authorities assess the hospital’s relevant costs during the approval process. These costs are then standardized based on the hospital’s case mix and compared with hospitals of similar service mandates.

We group patients into disease groups based on the primary diagnosis of ICD-10 and compute cost weights as the average between all patients in each group, ensuring a proportional representation of frequent treatments within categories. For base rates, we collected negotiated rates from cantonal authorities, computing cantonal averages over five years (Supplementary Material 1: Table S4). The final cost for each disease group is:

7 $$\begin{array}{l}{{\rm{Cost}}}_{({\rm{year}},{\rm{disease}}\,{\rm{group}},{\rm{canton}})}={\text{cost weight}}_{({\rm{year}},{\rm{disease}}\,{\rm{group}})}\\\times {\text{base rate}}_{({\rm{year}},{\rm{canton}})}\end{array}$$

Heat-attributable costs are calculated by multiplying attributable hospitalizations by average admission costs. The attributable number *AN* for historical periods is:

8 $$A{N}_{(x,year)}={a}_{(x,year)}\times A{F}_{(x,year)}$$

where *x* represents the combination of the disease group, the age group and the canton, and *a* is recorded hospital admissions during exposure days. The heat-attributable cost is then:

9 $${\text{Heat-Attributable Cost}}_{(x,year)}=A{N}_{(x,year)}\times {{\rm{Cost}}}_{(x)}$$

For future scenarios, we project heat-attributable hospitalizations that account for population growth and changing exposure frequency.

10 $$A{N}_{(x,{\rm{scenario}})}=A{F}_{(x,{\rm{scenario}})}\times {h}_{(x,{\rm{ref}})}\times growt{h}_{(x,{\rm{scenario}})}\times fre{q}_{({\rm{scenario}})}$$

where *h*_(*x*, ref)_ represents hospitalizations in the reference period during exposure days, *growth*_(*x*, scenario)_ is the age-specific population growth factor and *freq*_(scenario)_ is the ratio of projected to reference exposure days.

**Fig. 2:**
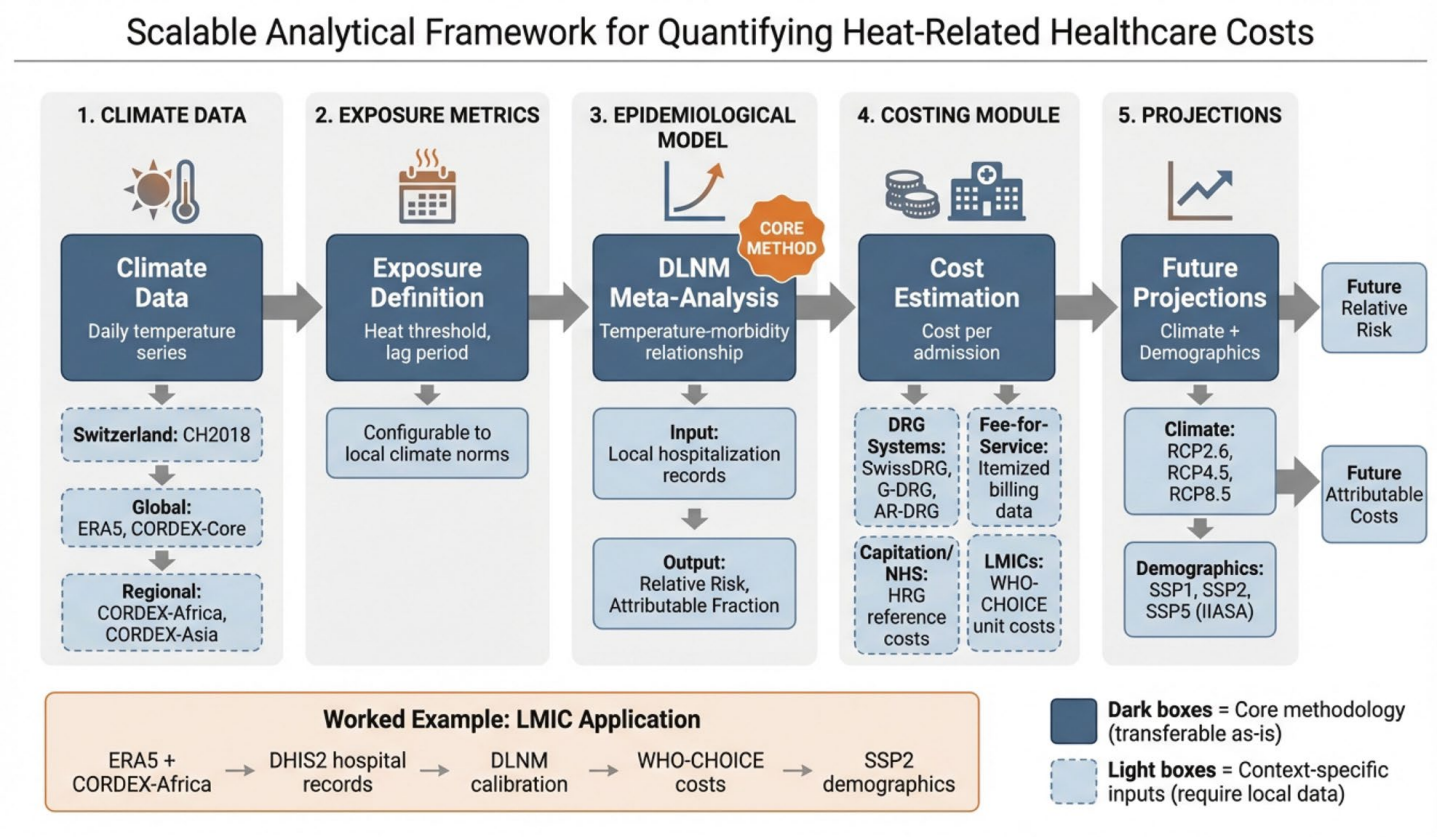
Schematic overview of the scalable analytical framework. The framework consists of five modular components: (1) climate data input, (2) exposure definition, (3) epidemiological modeling using DLNM, (4) cost estimation, and (5) future projections combining climate and demographic scenarios. Dark-shaded boxes indicate core methodology transferable across contexts; light-shaded boxes indicate context-specific inputs requiring local data. Examples of alternative data sources for different settings (e.g., LMIC applications using WHO-CHOICE costs and CORDEX projections) are shown beneath each module

Figure 2 presents a schematic overview of our analytical framework, designed to be scalable and transferable beyond the Swiss context. The modular structure allows researchers to substitute locally relevant climate data, epidemiological inputs, and costing methods, facilitating replication in settings ranging from high-income DRG-based systems to low- and middle-income countries (LMICs) using WHO-CHOICE unit costs.

## Results

### Historical relative risk analysis

**Fig. 3:**
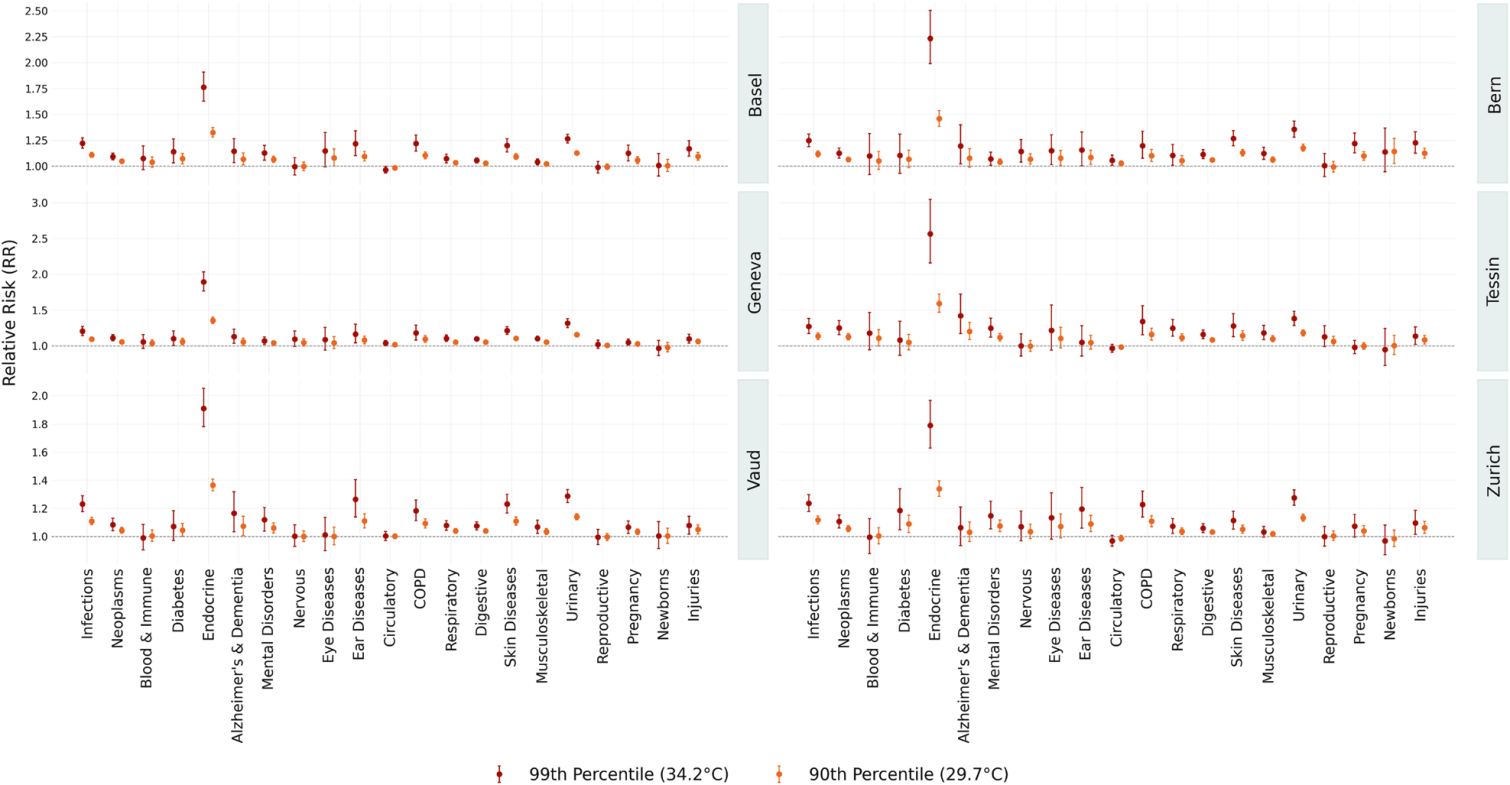
Cumulative RR for days with moderate and extreme heat. Canton-specific exposure-response functions are obtained by fitting a DLNM for each disease group and canton with a 7-day lag and then deriving the best linear unbiased prediction in a meta-regression model with cantonal random effects. All DLNM models are centered at the reference temperature of 23.3^∘^C, corresponding to the median warm-season’s maximum temperature across all cantons

Figure 3 presents cumulative RR estimates for extreme and moderate heat exposure on the day of exposure and the following week, in all disease groups and cantons. Extreme heat is defined as a daily maximum temperature of 34.2^∘^C (99th percentile), while moderate heat corresponds to 29.7^∘^C (90th percentile).

Endocrine disorders emerge as the most heat-sensitive group overall, with an average RR of 2.02 across cantons at the 99th percentile temperature. Next, we find urinary diseases (RR 1.31), infections (RR 1.23), COPD (RR 1.22), and skin disorders and diseases (RR 1.21). These results align with previous literature [20, 25] and reflect disease groups traditionally associated with heat stress. We do not observe a significant heat effect for conditions related to newborns or pregnancy, reproductive organs, blood and immune system, neurological, or musculoskeletal diseases. This was expected, as the analysis included all disease groups, including those not typically associated with heat sensitivity. Consistent with previous findings in Switzerland [24], we also observe a slightly negative association between heat and circulatory system conditions. Among cantons, Tessin (TI) shows the highest average RR (1.23 at 34.2^∘^C), followed by Basel (1.20), with endocrine risk in Tessin peaking as high as 2.56. When centering the RR at the cantonal median, Tessin (1.20 at 34.2^∘^C; 1.08 at 29.7^∘^C) and Basel (1.20 at 34.2^∘^C; 1.09 at 29.7^∘^C) remain the cantons with the highest average RR at both temperature thresholds. However, in most cases, 95% confidence intervals overlap, indicating that these differences are not statistically significant overall. Disease groups showing statistically higher RR in Tessin compared to specific cantons comprise endocrine, digestive, musculoskeletal, respiratory, and neoplasms.

At extreme heat, 59% of all disease-age–canton combinations show a statistically significant increase in risk compared to a typical warm-season day. Interestingly, at the moderate heat threshold of 29.7^∘^C, the share rises slightly to 62%, indicating that even less extreme heat exposure can significantly impact health. This highlights that heat-related risks extend well beyond extreme events, affecting a wide range of health outcomes across the population.

Supplementary Material 1: Fig. S1 shows 2022 cost weights by disease category and age group. Newborns and neoplasms carry the most expensive treatments, with a cost weight of 2.4 for neoplasm treatments in individuals aged 0–14, 1.67 for those aged 15–74, and 1.6 for newborn-related treatments. Among the least expensive treatments, we find ear diseases (0.44 for adults, 0.47 for the elderly), mental disorders (0.46 for adults), and diseases of the reproductive organs (0.54 for ages 0–14 and 0.75 for adults).

**Fig. 4:**
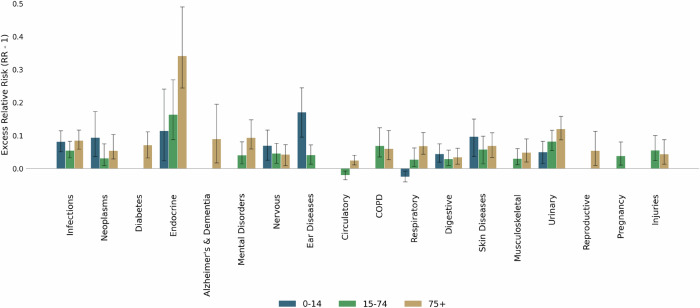
Average Relative Risk for different age groups

Figure 4 displays the average RR across all cantons during the historical period, stratified by age group. The highest risks are observed in the elderly (RR 1.081), followed closely by children (RR 1.077), with adults showing the lowest average RR (1.049). This confirms the well-established vulnerability of older individuals and children. Endocrine and metabolic diseases again stand out, with the 75+ group showing the highest RR (1.341), followed by adults (1.163) and children (1.114). In children, ear diseases show the strongest vulnerability to heat (RR 1.17), followed by skin conditions (1.096), neoplasms (1.093), and infections (1.081). Among adults, urinary diseases follow endocrine disorders (1.082), COPD (1.068), and skin diseases (1.057). However, no single disease category stands out strongly in this age group, and the overall effect is milder. In the elderly, after endocrine disorders, the highest risks are linked to urinary conditions (1.120), mental health disorders (1.093), Alzheimer’s and dementia (1.089), infections (1.085), and diabetes (1.070). Aging populations not only face a higher baseline burden but also a broader vulnerability spectrum.

### Future projections for relative risk

**Fig. 5:**
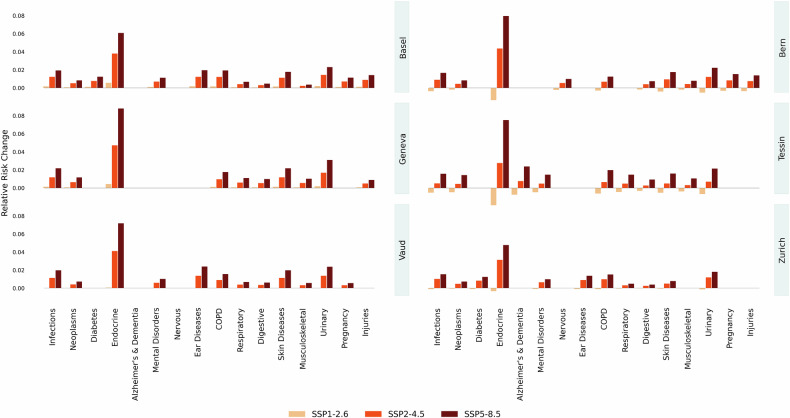
Projected heat relative risk difference in decade 2060–2069 compared to 2013–2022. We consider only disease-age-canton results that have a significant heat RR, namely, we discard those with a non-significant RR for temperatures above the heat threshold of 23.3^∘^C. Each bar represents the mean estimate for a given canton, scenario, and disease-age group. The corresponding version of the plot with confidence intervals is provided in Supplementary Material 1: Fig. S1

Consistent with the projected warming trends, heat-related health risks in Switzerland are expected to increase steadily between 2030 and 2070, particularly under the moderate (SSP2-RCP4.5) and high (SSP5-RCP8.5) emission scenarios. Figure 5 shows the projected increase in RR in the decade 2060–2069 compared to the past decade 2013–2022.

The largest increases are seen in adults aged 75 and older, especially for endocrine and metabolic disorders, diabetes, and mental health conditions, mirroring the disease categories already identified as vulnerable in the historical period. The canton of Geneva stands out as the most affected region, with an average increase in heat-related risk across all disease groups of 2.3% by 2070. This result remains unchanged when using the warm season’s cantonal median as reference temperature (average RR increase +2.1%), indicating that the projected risk is comparatively higher even when accounting for regional adaptation.

Under SSP1-RCP2.6, RR changes remain negligible or even slightly negative throughout the projected decades, reflecting the potential of strong mitigation efforts in limiting future heat-related health burdens. By contrast, under SSP5-RCP8.5, the heat-related RR increases gradually, reaching an average of +1.37% by the 2060s, more than 23 times the change projected under SSP1-RCP2.6. In line with the historical RR, the most pronounced effect is seen in endocrine disorders among the elderly, with a projected increase in heat-related risk peaking at +11% in Geneva. Other significantly affected groups include infections, mental disorders, urinary diseases in the elderly, and ear conditions in children. Urinary diseases in the elderly (75+) under SSP5-RCP8.5 rise from +0.15% in 2030 to +3.09% by 2070, while under the moderate emission scenario, they increase from +0.40% to +1.69%, still more than quadrupling over the same period. Similarly, infections and ear diseases in children and adolescents experience almost a 20-fold increase in the high emission scenario, reaching +2.34% and +5.5% respectively by 2070, and a four-fold increase in the moderate scenario. While some disease categories, such as reproductive and circulatory disorders, display smaller or less consistent trends, the overall picture points to a growing health burden in the absence of substantial emission reductions. Sensitivity analyses show that changing the reference period (e.g., 1998–2022 vs. 2018–2022) has only a minor effect on the projected change in RR. Differences across baselines remain within a few tenths of a percentage point. Earlier reference periods such as 1998–2022 and 2010–2019 yield even higher RR increases, while more recent baselines like 2013–2022 and 2018–2022, which already include extreme heat years, produce slightly attenuated projections.

Table 1 highlights the economic burden of heat exposure across six major Swiss cantons, namely Bern, Basel, Geneva, Ticino, Vaud, and Zurich, over a 10-year period from 2013 to 2022. On average, heat-related costs across these cantons amounted to CHF 20.6 million annually. Zurich experienced the greatest financial impact, with average yearly costs reaching CHF 4.7 million, while Geneva faced the lowest, at CHF 2.6 million.

**Table 1:** Average annual heat-attributable costs (2013–2022) by canton

Canton	Average Annual Cost (CHF)
Bern	4,106,907
Basel	3,009,085
Geneva	2,568,085
Ticino	3,441,362
Vaud	2,739,502
Zurich	4,689,121
**Total**	**20,554,061**

Table 2 presents the projected percentage changes in heat-attributable healthcare costs for the decade 2060–2069 compared to the baseline period 2013–2022, stratified by climate scenario, age group, and canton. The projections reveal striking age-related disparities and scenario-dependent escalation patterns. Under the low emission scenario (SSP1-RCP2.6), children and adults show mixed or slightly negative changes in most cantons, primarily due to demographic factors, while the elderly (75+) experience substantial increases, averaging 250% nationally. The moderate emission scenario (SSP2-RCP4.5) demonstrates consistently positive increases across all age groups, with children experiencing 45% higher costs on average, adults 26%, and the elderly facing nearly three-fold increases (284%). Under the high emission scenario (SSP5-RCP8.5), cost escalations become dramatic across all demographics: children face more than triple the baseline costs (204% increase), adults experience near-doubling (81% increase), and the elderly confront costs that are 4.6 times higher than historical levels (459% increase). Notably, Bern consistently shows the highest percentage increases across scenarios, while regional variations highlight differential vulnerability, with Geneva showing moderate increases and Ticino displaying relatively lower but still substantial cost escalations.

**Table 2:** Projected percentage change in heat-attributable costs (2060–2069 vs 2013–2022) by age group and canton

Scenario	Age Group	BE	BS	GE	TI	VD	ZH	Total
SSP1–RCP2.6	0–14	15%	− 29%	− 8%	− 17%	− 20%	− 16%	− 15%
	15–74	7%	− 16%	− 9%	− 15%	− 6%	− 9%	− 7%
	75+	290%	210%	245%	219%	268%	254%	250%
SSP2–RCP4.5	0–14	113%	35%	28%	22%	52%	39%	45%
	15–74	42%	18%	24%	9%	25%	29%	26%
	75+	329%	252%	269%	235%	310%	296%	284%
SSP5–RCP8.5	0–14	375%	187%	143%	162%	238%	177%	204%
	15–74	100%	67%	92%	63%	73%	84%	81%
	75+	520%	402%	455%	415%	490%	460%	459%

**Fig. 6:**
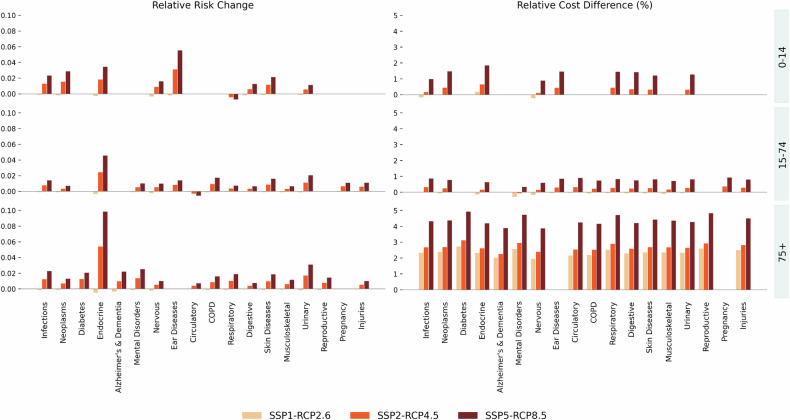
Projected change in heat RR and in heat-attributable healthcare costs across age groups and disease categories for the period 2060–2069 compared to 2013–2022, under different climate scenarios. **Left panel:** Y-axis shows the absolute change in RR (e.g., 0.02 indicates RR increased by 0.02 units). **Right panel:** Y-axis shows the relative cost increase as a percentage of baseline costs (e.g., 4.6 = 460% increase). Each bar represents the mean estimate for a given scenario, disease, and age group. Calculated costs are summed across cantons to obtain the total costs for each simulation and each climate path, then aggregated by SSP-RCP scenario. Calculated canton-specific RR values are averaged across cantons to obtain the Swiss average RR for each simulation and each climate path, then aggregated by SSP-RCP scenario. In the aggregation, we consider only disease-age-canton groups that have a significant heat RR, namely, we discard those with a non-significant RR for temperatures above 23.3^∘^C. The corresponding versions with empirical confidence intervals are shown in Supplementary Material 1: Figs. S4 and S5

Figure 6 shows projected changes in RR and heat-attributable healthcare costs at the national level, for the decade 2060–2069, compared to the baseline period 2013–2022. In line with rising RR, heat-related healthcare costs are expected to increase substantially across all scenarios, with sharper growth over time and under higher emission pathways. Under the moderate emissions scenario (SSP2-RCP4.5), additional annual costs across all cantons rise from CHF 8.1 million in the 2030s to CHF 30.1 million by the 2060s, around 3.7 times higher. Under SSP5-RCP8.5, excess heat-attributable healthcare costs are expected to be 2.5 times higher (+150%) than the heat-attributable costs in the reference period (equal to CHF 52 million per year) by the 2060s, more than five times the additional costs in 2030, and over six times higher than projections under SSP1-RCP2.6. Even under strong mitigation in SSP1-RCP2.6, annual costs are expected to triple, reaching CHF 23 million (+12% compared to the reference period) across the six cantons.

The elderly (75+) consistently generate the majority of these costs, often exceeding those of younger age groups by a factor of ten or more. For instance, during 2060–2069 under SSP5-RCP8.5, heat-attributable costs for the elderly are 5 times higher than in the reference period (cost increase equaling CHF 45 million across all cantons), compared to an increase of +80% (CHF 8.2 million) for those aged 15–74, and of 200% (approximately CHF 900,000) for children. This pattern is observed across nearly all disease categories, especially injuries, infections, endocrine and metabolic disorders, mental health conditions, and respiratory diseases. Injuries and external causes of hospitalizations emerge as the individual category with the largest increase in heat-attributable costs in absolute terms, reaching 12.1 million (+216%) across cantons and age groups by 2070 under SSP5-RCP8.5, and 6.8 million (+122%) in SSP2-RCP4.5. This category represents one of the most common causes of hospitalizations in the historical period, and its value is mostly driven by the comparatively high baseline fraction of patients. Among the disease groups with the highest relative cost increase, meaning the cost difference measured in terms of the historical heat-attributable cost, we find diabetes +492% (CHF 426,000 across significant cantons) under SSP5-RCP8.5, Alzheimer’s and dementia +389% (CHF 340,000), and respiratory diseases +346% (CHF 5 million). It is important to note that these cost estimates represent a conservative lower bound, capturing only direct hospitalization costs. The full economic burden, including outpatient care, emergency visits, and productivity losses, would be substantially higher (see the Discussion).

Supplementary Material 1: Fig. S6 shows the average annual costs and number of hospital admissions for each disease and age group during 2013-2022, summing across cantons. Injuries, circulatory diseases, and pregnancy and childbirth represent the largest cost shares, due to the high number of annual patients. The average hospital spending for urinary diseases in older adults during the baseline period was CHF 38.5 million annually across Switzerland. Of those, the cost attributable to temperatures above the heat threshold is estimated to be 2.5% (CHF 946,000). Under SSP5-RCP8.5, we estimate that climate change and aging will drive an additional CHF 3.3 million per year by 2060 and CHF 4 million by 2070, corresponding to an 8.5% and 10% increase in total urinary healthcare costs, respectively. This corresponds to a 4-fold increase in heat-attributable costs by 2070. Under the moderate emission scenario SSP2-RCP4.5, heat-related costs are expected to reach 3.5 million by the 2060s, corresponding to a 6.5% rise compared to the current annual costs. For the 15–74 group, the impact is less pronounced but not negligible, with a 1.5% increase in healthcare costs under SSP5-RCP8.5, reflecting slower demographic growth and lower sensitivity to heat. Similar trends appear in other heat-sensitive diseases such as diabetes, COPD, respiratory illnesses, and infections, each showing an approximate 4.5% rise in total annual costs by the 2060s under SSP5-RCP8.5. Reflecting the elevated RR observed, the greatest proportional increase is projected for endocrine and metabolic diseases in the elderly, with costs attributable to heat alone rising by 28% (CHF 3.5 million heat-attributable cost difference) under the high emissions scenario and 17% (CHF 2.2 million) under the moderate scenario by 2070.

To isolate the role of population growth in driving future healthcare costs, we computed projections in a ‘climate-only’ setting [12], where population size is held constant at baseline levels. The largest gap between the full and climate-only scenarios is observed under the low-emission pathway (SSP1-RCP2.6), as it combines minimal climate impact with a substantial demographic shift, particularly among the 75+ age group. Despite lower warming, this scenario still results in a significant cost increase due to aging alone, highlighting the crucial role of demographics in shaping future health burdens. Under the moderate and high emission pathways, climate change accounts for roughly 15–30% of the projected cost increases in the 75+ age group across future decades. For adults aged 15–74, the climate-only contribution is much higher, averaging around 80%, given their more stable population trends. In the 0–14 group, climate explains between 50% and 90% of the increase, depending on the decade and scenario. In SSP1-RCP2.6, the influence of climate is comparatively small for the elderly, accounting for approximately 5% of the total additional costs between 2050 and 2070. However, for adults and children, where the population is projected to decline or remain stable, climate is the main driver, explaining 85% of the increase in costs for adults and over 100% for children. Figure Supplementary Material 1: Fig. S15 illustrates the separate contribution of climate and demographic evolution to the heat-related costs over the future decades.

Finally, sensitivity to the choice of baseline period is minimal on the cost projections. Average and total cost changes remain effectively unchanged, with deviations within  ± 0.5% across all baselines tested. For instance, under SSP2-RCP4.5 in the 2060s, the average annual cost increase remains around CHF 109,000, and total national costs consistently reach approximately CHF 126 million regardless of baseline. Even under SSP5-RCP8.5, variation is negligible ( < 0.3%).

## Discussion

This analysis provides the first detailed, nationwide quantification of heat’s current and projected burden on hospitalizations and associated costs for Switzerland, considering a comprehensive range of health conditions and population groups. The findings underscore the urgent necessity for policymakers to implement and enhance heat adaptation measures, including heat-health warning systems, urban cooling initiatives, and protective protocols for vulnerable populations. By combining epidemiological modeling, cost estimation, and future projections, we provide a comprehensive framework to inform climate-resilient health system planning and policy in Switzerland and beyond.

A key strength of our analysis is the detailed stratification by age and disease category, coupled with future projections under various climate and socioeconomic scenarios. This granularity reveals specific vulnerabilities: the elderly (75+) and children (0–14) consistently exhibit higher RR for heat-related hospitalizations. For the elderly, endocrine/metabolic disorders, urinary diseases, mental health conditions, and diabetes are particularly pronounced. This aligns with physiological understanding, as older adults often have diminished thermoregulatory capacity, pre-existing chronic conditions, and may be on medications that interfere with heat adaptation [58]. Children, especially infants, have less developed thermoregulation and a greater surface-area-to-body-mass ratio [35], making them more susceptible to conditions like ear and skin infections, as our results indicate. The identification of endocrine and metabolic disorders as highly heat-sensitive across age groups is a notable finding, underscoring the systemic stress heat can impose. While some studies have focused on traditionally recognized heat-sensitive outcomes like renal or cardiovascular events [36], our broad approach uncovers less obvious but significant impacts.

The projection of substantial increases in heat-attributable healthcare costs, potentially reaching CHF 52 million annually by the 2060s under a high-emissions scenario (SSP5-RCP8.5), underscores the tangible economic burden of climate change. Importantly, even under strong mitigation (SSP1-RCP2.6), costs are projected to triple from the baseline, primarily driven by demographic aging. Our ‘climate-only’ analysis, which isolates the impact of warming from demographic shifts, reveals that while aging is a dominant driver of increased *total* healthcare demand, climate change significantly exacerbates this pressure. For the 75+ age group, climate change accounts for 15–30% of the projected cost increases in moderate to high emission scenarios, while for younger, more demographically stable populations, climate change becomes the primary driver of increased heat-related costs. This distinction is crucial for policy: while public health systems must prepare for an aging population regardless of climate change, targeted climate adaptation measures are essential to mitigate the *additional* burden imposed by rising temperatures.

The methodology employed, a two-stage DLNM meta-analysis, is a robust approach for time-series data in environmental epidemiology, allowing for the capture of non-linear and delayed temperature effects [43]. Integrating this with the SwissDRG system provides a data-driven approach to cost estimation. The use of a recent baseline period (2013–2022) is also a strength, as it reflects a period already experiencing notable warming, making future projections relative to this baseline more conservative and arguably more realistic for near-term planning.

Our estimated heat-attributable healthcare costs, averaging CHF 20.6 million annually across six major Swiss cantons during 2013–2022, are consistent with prior economic assessments of heat-related health impacts in Europe. For comparison [59], estimated the total economic burden of heatwaves in France at €25.5 billion over a five-year period (2015–2019), corresponding to approximately €5.1 billion annually, including €23.2 billion in mortality-related costs and €0.031 billion in morbidity costs. Although our analysis focuses solely on morbidity, specifically hospital admission costs, and therefore captures only a narrow component of the total health-related burden, these figures offer a consistent and policy-relevant estimate of the minimum economic impact of heat exposure.

However, these estimates must be interpreted as a conservative lower bound reflecting a payer-based perspective. By strictly quantifying SwissDRG reimbursements, our analysis excludes broader societal costs such as outpatient visits, emergency care, and, most notably, productivity losses. To contextualize this underestimation, recent findings indicate that heat-related labor productivity losses in Switzerland are estimated at approximately CHF 665 million annually, over 30 times higher than the direct hospital costs identified in this study [15]. Therefore, while our results provide a robust baseline for health-system planning, they capture only a fraction of the total economic burden, which would be much higher when comprehensive cost-of-illness frameworks are applied.

More specifically, a comprehensive cost-of-illness approach, as outlined in WHO-CHOICE guidelines [60], would additionally encompass: (i) direct medical costs beyond hospitalization, including outpatient visits, pharmaceuticals, and rehabilitation; (ii) direct non-medical costs such as patient transportation and informal caregiving; (iii) indirect costs including productivity losses from absenteeism and premature mortality; and (iv) intangible costs reflecting reduced quality of life and suffering [32]. The inclusion of these components would substantially increase the estimated burden, reinforcing that our hospital-based figures represent a conservative lower bound of the true societal cost of heat exposure.

To provide context for policy interpretation and allow readers to estimate the likely magnitude of omitted costs, we draw on literature-based multipliers. Studies suggest that for every heat-related hospitalization, there are approximately 2–4 additional emergency department (ED) visits that do not result in admission [61, 62], and that heat-related primary care consultations may be 5–10 times more frequent than hospitalizations, albeit at substantially lower per-visit costs [28]. Furthermore, comprehensive cost-of-illness studies indicate that inpatient costs typically represent only 40–60% of total direct medical expenditures [32]. Applying these multipliers, our estimated CHF 20.6 million annual hospital burden likely corresponds to approximately CHF 35–50 million in total direct healthcare costs. This figure remains modest compared to indirect costs: heat-related labor productivity losses in Switzerland alone are estimated at CHF 665 million annually [15]. For further comparison, a recent assessment of heatwave impacts in France estimated mortality-related welfare losses at €23.2 billion over five years, compared to €31 million for direct morbidity costs [59], illustrating that mortality costs can exceed morbidity costs by orders of magnitude. Taken together, these comparisons underscore that our hospital-based estimates represent a small fraction of the total societal burden of heat exposure.

Despite representing a fraction of the total societal cost, the projected burden on hospital expenditures remains significant. In our case, projected annual heat-related hospital costs across all cantons under the high-emission scenario (SSP5-RCP8.5) reach CHF 52 million by the 2060s, representing a 150% increase from baseline. This corresponds to approximately 0.5–1.2% of total annual hospital expenditure in Switzerland (based on recent Swiss Federal Office of Public Health estimates of CHF 35–40 billion annually), a proportion that underscores the growing significance of climate-sensitive health costs. The magnitude and trajectory of these costs appear reasonable given demographic aging, baseline hospitalization patterns, and increasing heat exposure. Furthermore, our projections are grounded in disease- and age-specific RR estimates derived from historical data and validated through multiple sensitivity analyses, supporting the robustness of the findings.

These estimates are further supported by other studies. For instance [37], estimated that heat-attributable morbidity costs in major U.S. cities could exceed USD 40 million per year in extreme cases. While absolute values differ by country, healthcare system, and climate baseline, the order of magnitude is consistent with our projections when adjusted for population and coverage. Similarly, WHO assessments [63] stress that morbidity costs are often underestimated and should be included in climate-health policy frameworks. Our analysis addresses this gap by focusing explicitly on hospitalization expenditures, which are more directly attributable and measurable than mortality or well-being costs.

The reliance on national-level SSP demographic projections, assuming uniform cantonal growth, is another simplification due to the unavailability of Swiss-specific SSP-CH scenarios at the time of writing. Cantonal-level demographic trends could refine these projections. Lastly, by focusing on direct hospital costs, we do not capture the full economic impact, which would include indirect costs (e.g., productivity losses, informal care) and costs associated with primary care visits or outpatient treatments not captured in our dataset. A full cost-of-illness approach, as employed by [32], would provide a more holistic economic picture.

While this study focuses on Switzerland, the analytical framework is designed to be modular and transferable to other contexts (Fig. 2). Each component can be adapted to local conditions: climate inputs can be substituted with globally available datasets such as ERA5 reanalysis for historical data or CORDEX-Core regional projections for future scenarios; the DLNM epidemiological approach is inherently flexible and has been successfully applied across diverse climates and populations, requiring only local temperature and hospitalization records for recalibration. The costing module is the most context-dependent component but can be adapted to various health system structures. In DRG-based systems (e.g., Germany, Australia, France), local DRG tariffs can be directly substituted for SwissDRG. In fee-for-service systems (e.g., United States), itemized billing data or average charges per admission can be used. In capitation or global budget systems (e.g., UK National Health Service), Healthcare Resource Group (HRG) reference costs provide a comparable metric. For LMIC settings lacking DRG infrastructure, WHO-CHOICE unit costs (e.g., cost per bed-day) offer standardized proxies that enable cross-country comparisons [60]. As a worked example, a researcher in Sub-Saharan Africa could implement this framework by: (i) obtaining daily temperature from ERA5 and future projections from CORDEX-Africa; (ii) collecting hospital admission records from national health information systems such as DHIS2; (iii) fitting location-specific DLNM exposure-response functions; (iv) applying WHO-CHOICE unit costs for the relevant WHO region; and (v) using IIASA SSP demographic projections for the country of interest. This modularity ensures the framework can serve as a scalable template for quantifying heat-health costs globally, beyond the specificities of the Swiss context.

However, several limitations warrant consideration. Our projections rely on CH2018 climate scenarios, which are based on CMIP5 models. Recent evidence indicates that temperatures over Western and Central Europe have risen more sharply since 1980 than projected in these models, partly due to reduced aerosol concentrations (the “brightening effect”) that were not fully captured [64, 65]. The recently released CH2025 scenarios better account for these effects. According to NCCS, while CH2018’s core qualitative statements remain valid, quantitative changes are expected to be more pronounced. Consequently, our projections likely represent a conservative estimate of future heat-health impacts; future work using CH2025 may reveal even greater burdens than presented here. The CH2018 climate data, while remaining valid for Switzerland, may not fully capture microclimatic variations, particularly urban heat island (UHI) effects in densely populated cantons. UHIs can significantly elevate local temperatures beyond regional averages, which may lead to an underestimation of exposure and impacts in urban centers where a large proportion of the population resides [12, 66]. Future research could benefit from integrating higher-resolution temperature data or UHI-specific adjustments. We acknowledge that our framing of results as a “conservative lower bound” may appear in tension with the inclusion of the high-emission SSP5-RCP8.5 scenario. To clarify: the “lower bound” framing refers exclusively to the scope of costs captured namely, direct hospital reimbursements which represent only a fraction of the total economic burden of heat on health (excluding outpatient care, emergency visits, productivity losses, and intangible costs). It does not refer to the climate scenarios themselves. The SSP5-RCP8.5 scenario is included not as a prediction, but as a high-end risk benchmark consistent with established practice in climate impact assessment, and is the highest-emission scenario available in the CH2018 projections used in this study. Even under this scenario, the costs we report remain a lower bound of the true health-system burden, precisely because they capture only hospital reimbursements. We recognize that future work using the recently released CH2025 climate scenarios, which better account for observed warming trends in Central Europe, would provide updated and potentially higher impact estimates. Framing our results in terms of hospital reimbursements rather than total societal costs ensures transparency about what is measured, while the literature-based multipliers provided in the Discussion allow readers and policymakers to approximate the broader economic burden.

Our definition of heat exposure, using a fixed national warm-season median (23.3^∘^C) as the reference, provides consistency across disease groups and an intuitive interpretation of “hotter-than-average” days. However, this differs from approaches using disease-specific MMTs, which might capture impacts at lower temperatures for certain conditions. While our approach focuses on deviations from typical warm conditions, it might underestimate the burden if significant health effects occur at temperatures below this median but above an individual’s or condition’s optimal temperature. The sensitivity analysis using MMTs (shown in the Supplementary Material 1) indeed suggests different risk profiles, and this choice warrants careful consideration in interpreting the magnitude of effects.

Furthermore, our use of average DRG cost weights may underestimate the true cost of heat-related admissions. Evidence suggests that heat-related cases are often more severe than average, with higher intensive care unit (ICU) admission rates and longer lengths of stay [28, 61]. As DRG tariffs reflect average resource consumption within a diagnostic group, they may not fully capture the marginal intensity of heat-attributable hospitalizations. We note, however, that SwissDRG cost weights are updated annually based on actual hospital cost accounting data, which partially captures rising treatment intensities over time, though not the marginal intensity of specific extreme heat events.

To assess the sensitivity of our cost estimates to this assumption, we applied severity multipliers reflecting the potential additional resource intensity of heat-related admissions. Based on literature suggesting 10–30% longer length of stay and higher complication rates for heat-related cases [28, 61], we tested multipliers of 1.1, 1.2, and 1.3 applied to our baseline estimates. Under these assumptions, the average annual heat-attributable cost (2013–2022) would increase from CHF 20.6 million at baseline to CHF 22.7 million (+10%), CHF 24.7 million (+20%), or CHF 26.8 million (+30%). This sensitivity analysis indicates that our baseline estimates may underestimate true costs by CHF 2–6 million annually, reinforcing that the figures presented represent a conservative lower bound. It is important to note that our cost estimates are based on SwissDRG reimbursement tariffs, which reflect standardized payments to hospitals rather than actual resource consumption. Reimbursement-based cost estimates may diverge from true economic costs in both directions: they may underestimate costs for complex heat-related cases requiring above-average resources (e.g., prolonged ICU stays), while potentially overestimating costs for routine cases where actual resource use falls below the tariff amount. Despite this limitation, SwissDRG tariffs represent the most systematic and nationally standardized measure of hospitalization costs available in Switzerland and are widely used in health economic analyses.

Additionally, to account for heterogeneity in hospital base rates across different hospital types within cantons, we conducted a sensitivity analysis applying  ± 15% bands to our cost estimates. Negotiated base rates vary across hospitals within the same canton, for example, ranging from approximately CHF 9,500 to CHF 11,200 in Zurich, reflecting differences between tertiary university hospitals and smaller regional facilities. Applying this variation to our baseline estimate yields a plausible range of CHF 17.5–23.7 million annually, compared to our central estimate of CHF 20.6 million. This range remains consistent with our interpretation of substantial and growing heat-related healthcare costs.

The study did not explicitly model the influence of environmental co-factors like air pollution (e.g., ozone, PM2.5) or humidity, which can interact with heat to exacerbate health risks [19, 67]. While some studies suggest temperature effects are largely independent of certain pollutants for mortality [58], their combined impact on morbidity warrants further investigation, especially given that heatwaves often coincide with periods of poor air quality.

Furthermore, our projections do not incorporate future adaptation measures or changes in population vulnerability beyond demographic shifts. As societies adapt through improved early warning systems, urban greening, building retrofits, and behavioral changes, the realized health burden may be lower than projected [55]. However, quantifying the effectiveness and uptake of such measures is complex and model-dependent. Our estimates should therefore be seen as projections under current vulnerability levels, highlighting the potential burden *without* enhanced adaptation. Similarly, we assume future hospitalization rates per capita (for a given age group) remain constant, which might overestimate demand if, for example, improvements in healthy aging reduce hospital admission rates among the elderly. Recent evidence from Switzerland indicates that heat-related mortality risk has already declined in some regions following the implementation of heat-health action plans (HHAPs), suggesting that structured adaptation measures can effectively reduce vulnerability over time [68].

The reliance on national-level SSP demographic projections, assuming uniform cantonal growth, is another simplification due to the unavailability of Swiss-specific SSP-CH scenarios at the time of writing. Cantonal-level demographic trends could refine these projections. Lastly, by focusing on direct hospital costs, we do not capture the full economic impact, which would include indirect costs (e.g., productivity losses, informal care) and costs associated with primary care visits or outpatient treatments not captured in our dataset. A full cost-of-illness approach, as employed by [32], would provide a more holistic economic picture.

Despite these limitations, this study provides crucial evidence for Swiss policymakers and healthcare planners. The findings highlight the urgent need for proactive measures, including strengthening heat-health action plans, investing in urban cooling strategies, and enhancing climate mitigation efforts to protect public health and manage escalating healthcare expenditures in the face of a warming climate.

## Conclusions

This study provides the first comprehensive quantification of heat-related morbidity and associated healthcare costs in Switzerland, addressing a critical gap in understanding the full health burden of climate change beyond mortality impacts. Our findings demonstrate that even in a high-income, temperate nation with a well-resourced healthcare system, climate change is already imposing a measurable health burden, which is projected to escalate considerably in the coming decades.

Heat exposure significantly increases hospital admissions for a range of conditions, most notably endocrine/metabolic disorders, urinary diseases, infections, and, in children, ear and skin conditions; even moderate heat (29.7^∘^C) affects 62% of disease-age-canton combinations. The elderly (75+) and children (0–14) are the most vulnerable age groups, with regional variations showing Geneva and Ticino among the most affected areas. Future projections indicate substantial increases in heat-attributable healthcare costs, with estimates reaching 2.5-fold increases under high-emission scenarios and 3.7-fold increases under moderate scenarios by the 2060s. While demographic aging is a primary driver of overall healthcare demand, climate change significantly amplifies this burden. Even under strong climate mitigation scenarios, heat-related healthcare costs are projected to triple due to committed warming and population aging, underscoring the dual need for both mitigation and adaptation.

The evidence presented issues an urgent call for proactive, nationwide policy. These financial figures, representing a conservative lower bound by excluding outpatient care, pharmaceutical expenses, and indirect societal costs like productivity loss, provide a robust economic rationale for investment in adaptation. Strengthening heat-health action plans, investing in urban cooling, and enhancing protective protocols are critical measures. This research provides the critical economic evidence base needed to justify and design these strategies, aiming to safeguard public health and fortify the resilience of the Swiss healthcare system in a rapidly warming world.

## Supplementary information

Supplementary material 1: **Fig. S1**: Swiss DRG cost weights by disease and age group (2022); **Fig. S2**: Relative risk for different age groups by canton; **Fig. S3**: Projected heat relative risk difference in 2060–2069, with confidence intervals; **Fig. S4**: Projected change in heat-attributable healthcare costs, with confidence intervals; **Fig. S5**: Projected change in heat relative risk by age and disease, with confidence intervals; **Fig. S6**: Average annual hospitalization costs and admissions (2013–2022); Figs. **S7–S12**: Exposure-response curves by canton (Bern, Basel, Geneva, Ticino, Vaud, Zürich); **Fig. S13**: Demographic composition under different SSP scenarios; **Fig. S14**: Difference between SSP-RCP and climate-only cost projections (2060–2069); **Fig. S15**: Evolution of SSP-RCP vs. climate-only cost difference over future decades; **Fig. S16**: Sensitivity analysis of model configurations; **Fig. S17**: Comparison of temperature variables (Tmin, Tmean, Tmax); **Fig. S18**: Cost weight distribution across disease groups (2022); **Fig. S19**: Evolution of cost weights by disease group (2012–2022); **Fig. S20**: Relative risk difference using cantonal vs. Swiss-wide median temperature; **Figs. S21–S26**: Exposure-response curves with disease-specific MMT by canton (Bern, Basel, Geneva, Ticino, Vaud, Zürich); **Table S1**: Summary of temperature simulation models (CH2018); **Table S2**: Disease group definitions with ICD-10 and Swiss DRG mappings; **Table S3**: Cantonal temperature statistics for the warm season (May–September); **Table S4**: Hospital-specific base rates by canton (2012–2026); **Table S5**: Projected difference in heat-attributable healthcare costs (2060–2069 vs. 2013–2022); **Table S6**: Estimated annual heat-attributable healthcare costs (2013–2022)

## Data Availability

Hospital admission data were obtained under contract No. 240448 with the Swiss Federal Statistical Office (BFS) and cannot be publicly shared due to data protection requirements. Researchers may request access from BFS under comparable contractual conditions; requests can be submitted via https://www.bfs.admin.ch/bfs/en/home/services/contact.html. The CH2018 climate scenario data are publicly available at https://www.nccs.admin.ch/nccs/en/home/climate-change-and-impacts/swiss-climate-change-scenarios.html. SwissDRG tariff data are publicly available at https://www.swissdrg.org. All analytical code and shareable data supporting the findings of this study are available at https://github.com/saeedashraf/Cost-ClimateChange-Health[69].

## Abbreviations

AF: Attributable Fraction
BAFU: Swiss Federal Office for the Environment
BFS: Swiss Federal Statistical Office
BLUP: Best Linear Unbiased Prediction
CHF: Swiss Francs
COPD: Chronic Obstructive Pulmonary Disease
DLNM: Distributed Lag Non-Linear Model
DRG: Diagnosis-Related Group
ED: Emergency Department
EHA: Emergency Hospital Admission
GDP: Gross Domestic Product
HHAP: Heat-Health Action Plan
HRG: Healthcare Resource Group
ICD-10: International Classification of Diseases, 10th Revision
ICU: Intensive Care Unit
IIASA: International Institute for Applied Systems Analysis
LMIC: Low- and Middle-Income Country
MMT: Minimum Morbidity Temperature
NCAR: National Center for Atmospheric Research
NCCS: National Centre for Climate Services
RCP: Representative Concentration Pathway
RR: Relative Risk
SSP: Shared Socioeconomic Pathway
SwissDRG: Swiss Diagnosis-Related Groups
UHI: Urban Heat Island
USD: United States Dollars
WHO: World Health Organization
WMO: World Meteorological Organization
