# Supplementary material for: Quantifying the financial burden of heat-related hospital admissions in Switzerland under a changing climate: A scalable analytical framework
Source: BMC Glob Public Health. 2026 May 28;4:52. doi: 10.1186/s44263-026-00275-w (PMC13217927; doi:10.1186/s44263-026-00275-w)
Supplement: Supplementary file 1 — Supplementary material 1: Fig. S1: Swiss DRG cost weights by disease and age group (2022); Fig. S2: Relative risk for different age groups by canton; Fig. S3: Projected heat relative risk difference in 2060–2069, with confidence intervals; Fig. S4: Projected change in heat-attributable healthcare costs, with confidence intervals; Fig. S5: Projected change in heat relative risk by age and disease, with confidence intervals; Fig. S6: Average annual hospitalization costs and admissions (2013–2022); Figs. S7–S12: Exposure-response curves by canton (Bern, Basel, Geneva, Ticino, Vaud, Zürich); Fig. S13: Demographic composition under different SSP scenarios; Fig. S14: Difference between SSP-RCP and climate-only cost projections (2060–2069); Fig. S15: Evolution of SSP-RCP vs. climate-only cost difference over future decades; Fig. S16: Sensitivity analysis of model configurations; Fig. S17: Comparison of temperature variables (Tmin, Tmean, Tmax); Fig. S18: Cost weight distribution across disease groups (2022); Fig. S19: Evolution of cost weights by disease group (2012–2022); Fig. S20: Relative risk difference using cantonal vs. Swiss-wide median temperature; Figs. S21–S26: Exposure-response curves with disease-specific MMT by canton (Bern, Basel, Geneva, Ticino, Vaud, Zürich); Table S1: Summary of temperature simulation models (CH2018); Table S2: Disease group definitions with ICD-10 and Swiss DRG mappings; Table S3: Cantonal temperature statistics for the warm season (May–September); Table S4: Hospital-specific base rates by canton (2012–2026); Table S5: Projected difference in heat-attributable healthcare costs (2060–2069 vs. 2013–2022); Table S6: Estimated annual heat-attributable healthcare costs (2013–2022) [file 44263_2026_275_MOESM1_ESM.pdf]

Supplementary Material 1

Quantifying the Financial Burden of Heat-Related Hospital Admissions in Switzerland under a Changing Climate: A Scalable Analytical Framework

Supplementary Materials 1

Overview

This Supplementary Material accompanies the manuscript "Quantifying the Financial Burden of Heat-Related Hospital Admissions in Switzerland under a Changing Climate: A Scalable Analytical Framework." It contains 26 supplementary figures (Figs. S1–S26) and 6 supplementary tables (Tables S1–S6) organized into seven sections: (1) heat-related risks and costs, (2) relative risk curves by canton, (3) socioeconomic influence on climate projections, (4) disease group definitions, (5) sensitivity analyses, (6) results with alternative reference temperatures, and (7) hospital base rates and cost data.

Table of Contents

Figures

- Fig. S1: Swiss DRG cost weights by disease and age group (2022)
- Fig. S2: Relative risk for different age groups by canton
- Fig. S3: Projected heat relative risk difference in 2060–2069, with confidence intervals
- Fig. S4: Projected change in heat-attributable healthcare costs, with confidence intervals
- Fig. S5: Projected change in heat relative risk by age and disease, with confidence intervals
- Fig. S6: Average annual hospitalization costs and admissions (2013–2022)
- Figs. S7–S12: Exposure-response curves by canton (Bern, Basel, Geneva, Ticino, Vaud, Zürich)
- Fig. S13: Demographic composition under different SSP scenarios
- Fig. S14: Difference between SSP-RCP and climate-only cost projections (2060–2069)
- Fig. S15: Evolution of SSP-RCP vs. climate-only cost difference over future decades
- Fig. S16: Sensitivity analysis of model configurations
- Fig. S17: Comparison of temperature variables (T_{min}, T_{mean}, T_{max})
- Fig. S18: Cost weight distribution across disease groups (2022)
- Fig. S19: Evolution of cost weights by disease group (2012–2022)
- Fig. S20: Relative risk difference using cantonal vs. Swiss-wide median temperature
- Figs. S21–S26: Exposure-response curves with disease-specific MMT by canton (Bern, Basel, Geneva, Ticino, Vaud, Zürich)

Tables

- Table S1: Summary of temperature simulation models (CH2018)
- Table S2: Disease group definitions with ICD-10 and Swiss DRG mappings
- Table S3: Cantonal temperature statistics for the warm season (May–September)
- Table S4: Hospital-specific base rates by canton (2012–2026)
- Table S5: Projected difference in heat-attributable healthcare costs (2060–2069 vs. 2013–2022)
- Table S6: Estimated annual heat-attributable healthcare costs (2013–2022)

Heat related risks and costs

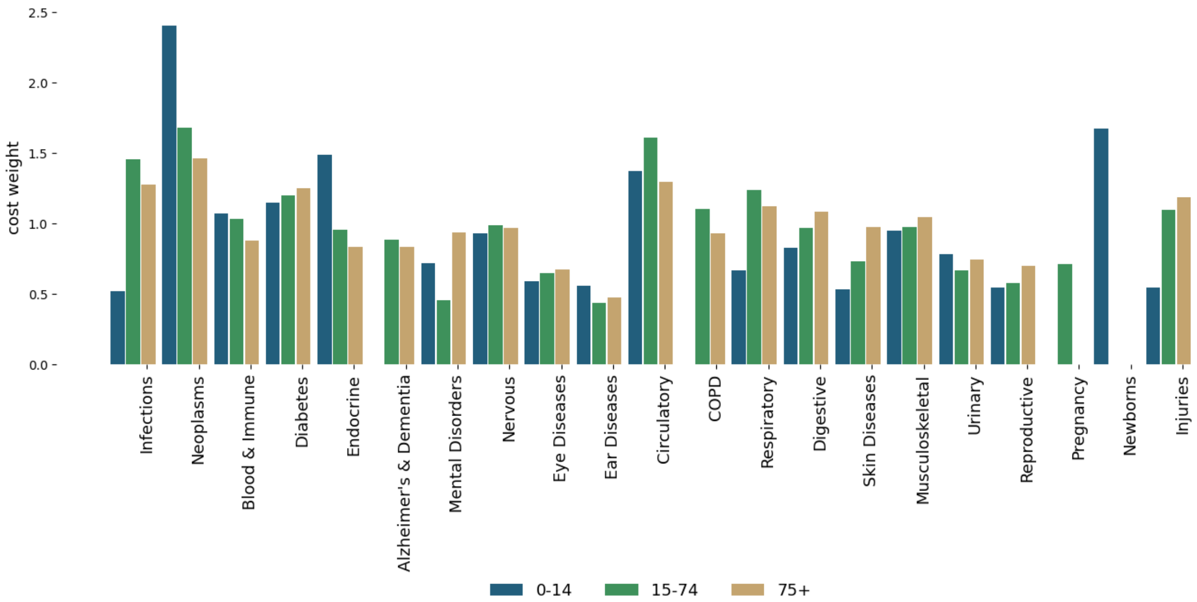

Fig. S1: **Swiss DRG cost weights by disease and age group (2022)**
Cost weights for each disease and age group are obtained by first assigning to each patient in the sample the corresponding cost of treatment depending on their Swiss DRG code in the dataset. This is done for each year in 2013-2022 by mapping the cost weights published by Swiss DRG for that year to the Swiss DRG code in the sample. Then we group patients by year, age group, and disease group, and calculate the average cost weight by taking the average of the cost weights for each stratum. We choose to use the latest cost weights available (year 2022) as a proxy for future cost weights.

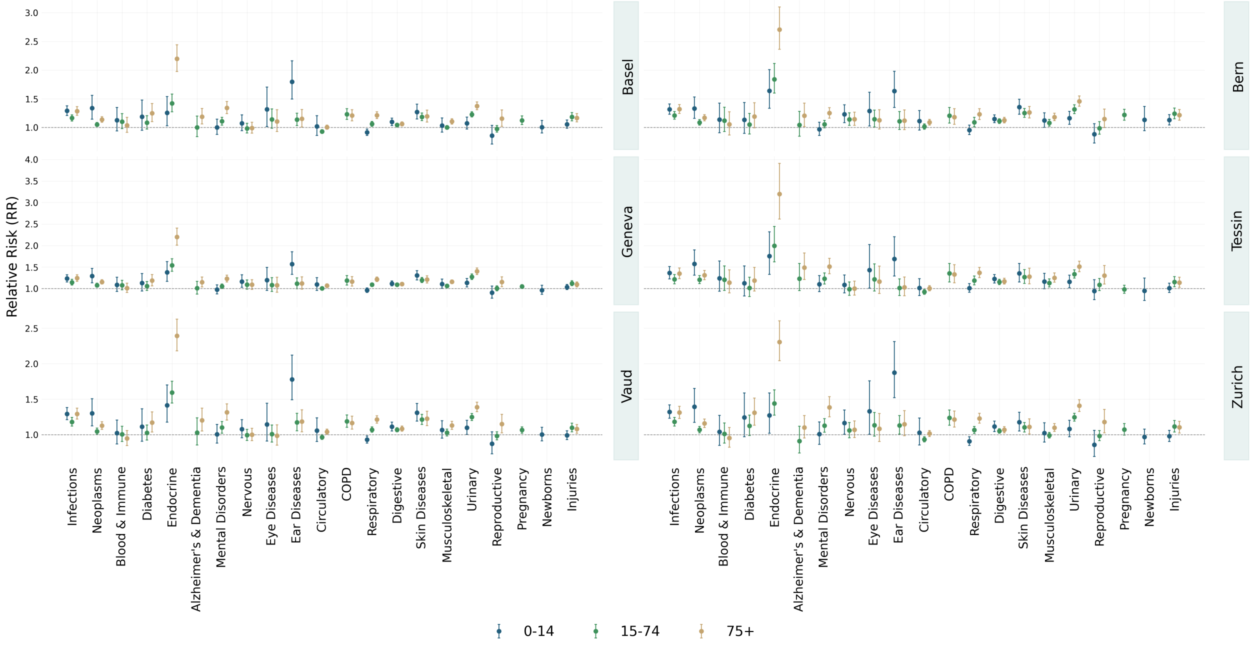

Fig. S2: **Relative Risk for different age groups.** Average Relative Risk for different age groups by canton during the reference period 2013-2022

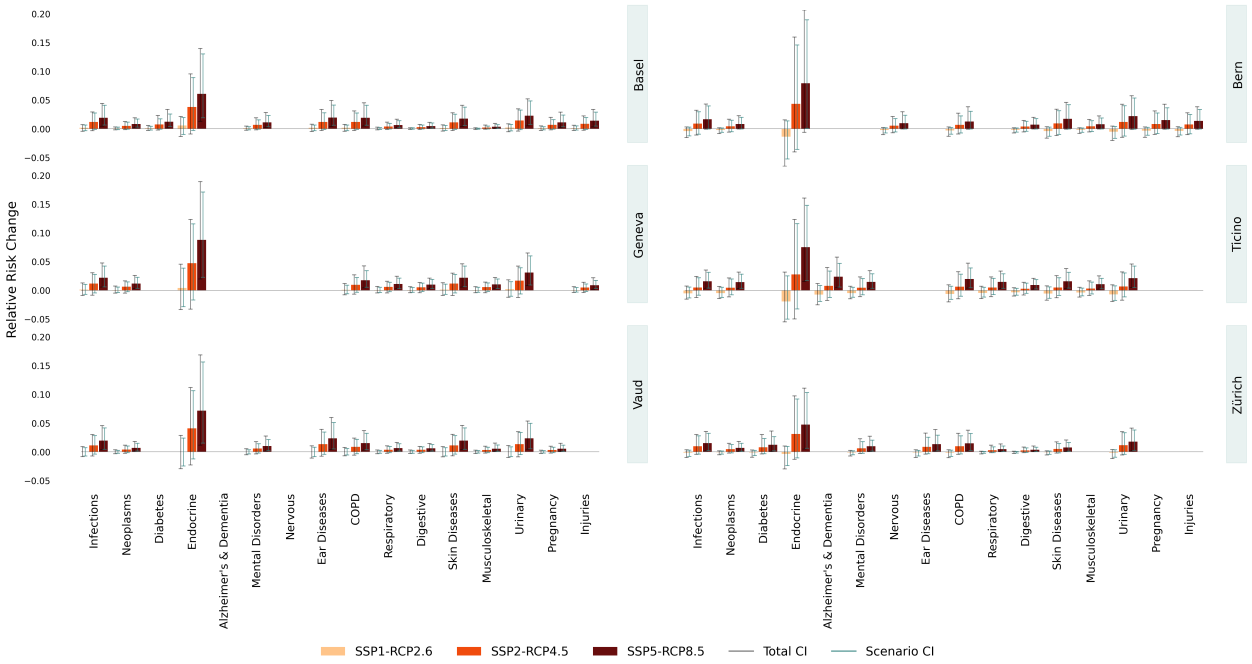

Fig. S3: **Projected Heat Relative Risk Difference in Decade 2060-2069 compared to 2013-2022.** Black whiskers show the total uncertainty (95% empirical confidence interval) including both within-scenario variability given by the uncertainty in the relative risk estimates and between-scenario variability. Blue whiskers represent the climate-only uncertainty, reflecting only the spread across different climate paths in the same SSP-RCP.

Shaded bands represent 95% confidence intervals, incorporating both epidemiological uncertainty (from 500 Monte Carlo simulations of the exposure-response unction) and climate model uncertainty (from the spread across 68 simulations).

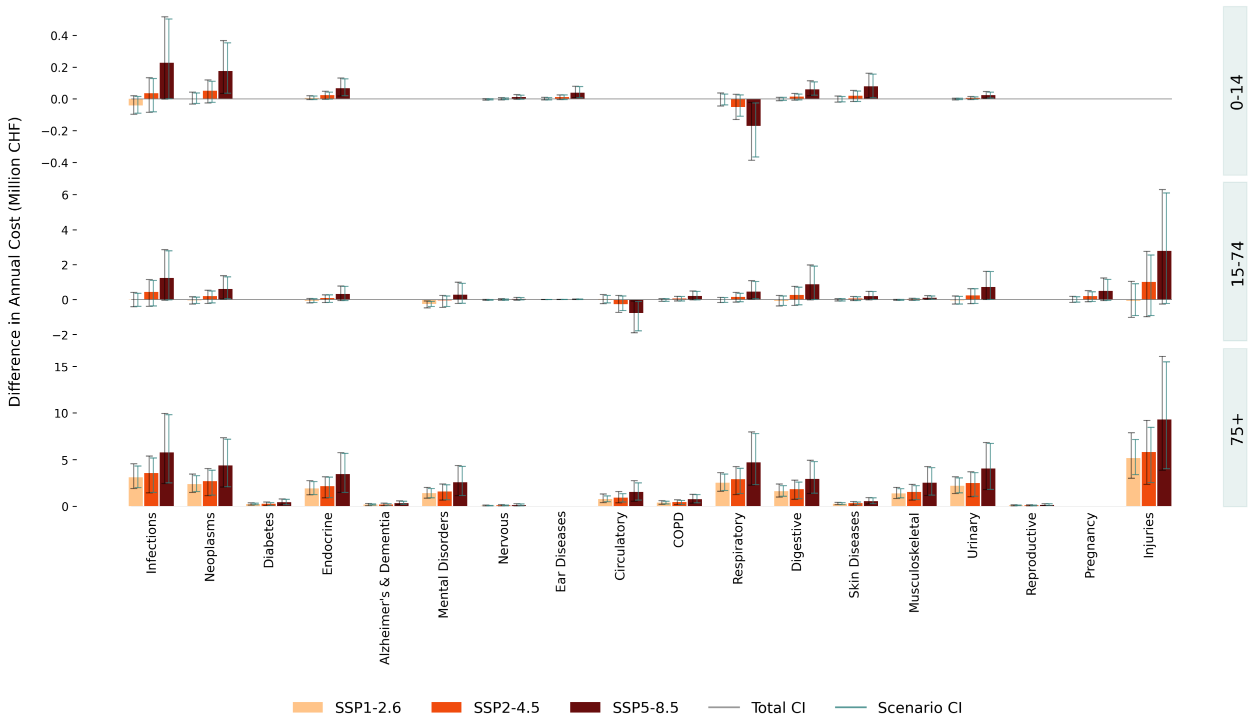

Fig. S4: **Projected change in heat-attributable healthcare costs across age groups and disease categories for the period 2060–2069 compared to 2013–2022, under different climate scenarios.** Each bar represents the mean estimate for a given scenario, disease, and age group. Calculated costs are summed across cantons to obtain the total costs for each simulation and each climate path, then aggregated by SSP-RCP scenario. Black whiskers show the total uncertainty (95% confidence interval) including both within-scenario variability given by the uncertainty in the relative risk estimates and between-scenario variability. Blue whiskers represent the climate-only uncertainty, reflecting only the spread across different climate paths in the same SSP-RCP.

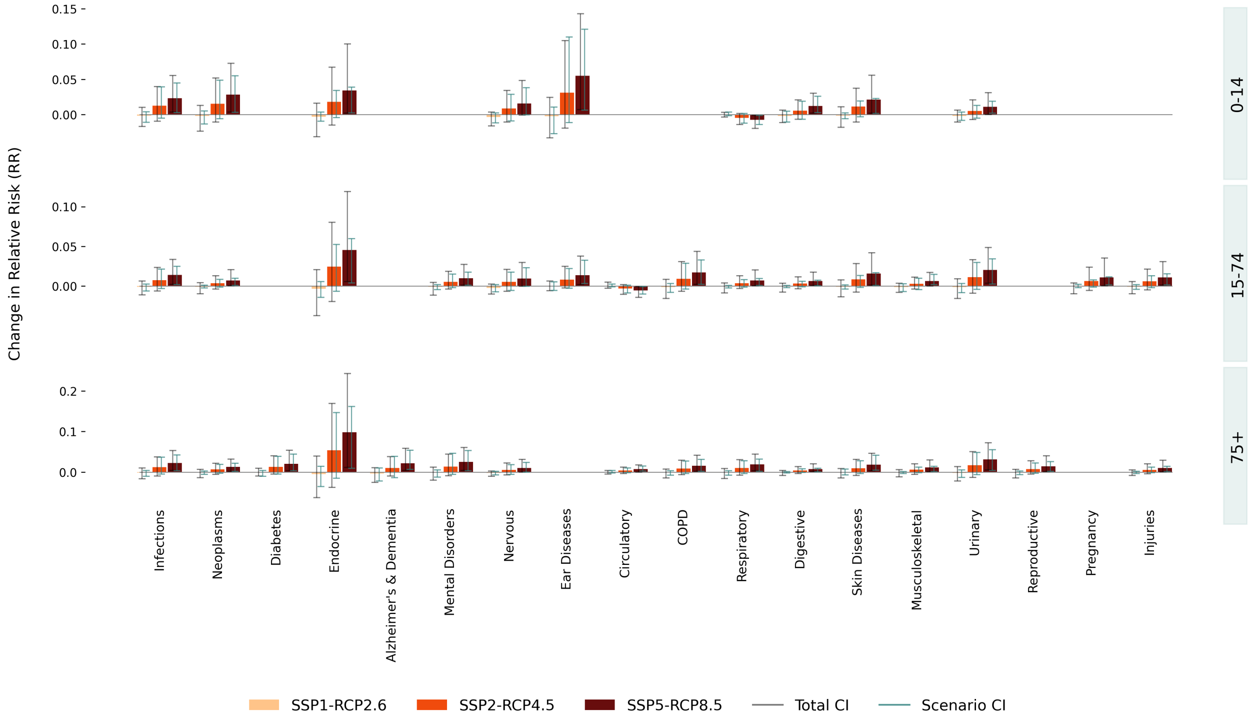

Fig. S5: **Projected change in heat relative risk across age groups and disease categories for the period 2060–2069 compared to 2013–2022, under different climate scenarios.** Calculated canton-specific relative risks are averaged across cantons to obtain the Swiss average relative risk for each simulation and each climate path, then aggregated by SSP-RCP scenario. In the aggregation, we consider only disease-age-canton results that have a significant heat relative risk, namely, we discard those with a non-significant relative risk for temperatures above 23.3. Each bar represents the mean estimate for a given scenario and disease-age group. Black whiskers show the total uncertainty (95% confidence interval) including both within-scenario variability given by the uncertainty in the relative risk estimates and between-scenario variability. Blue whiskers represent the climate-only uncertainty, reflecting only the spread across different climate paths in the same SSP-RCP.

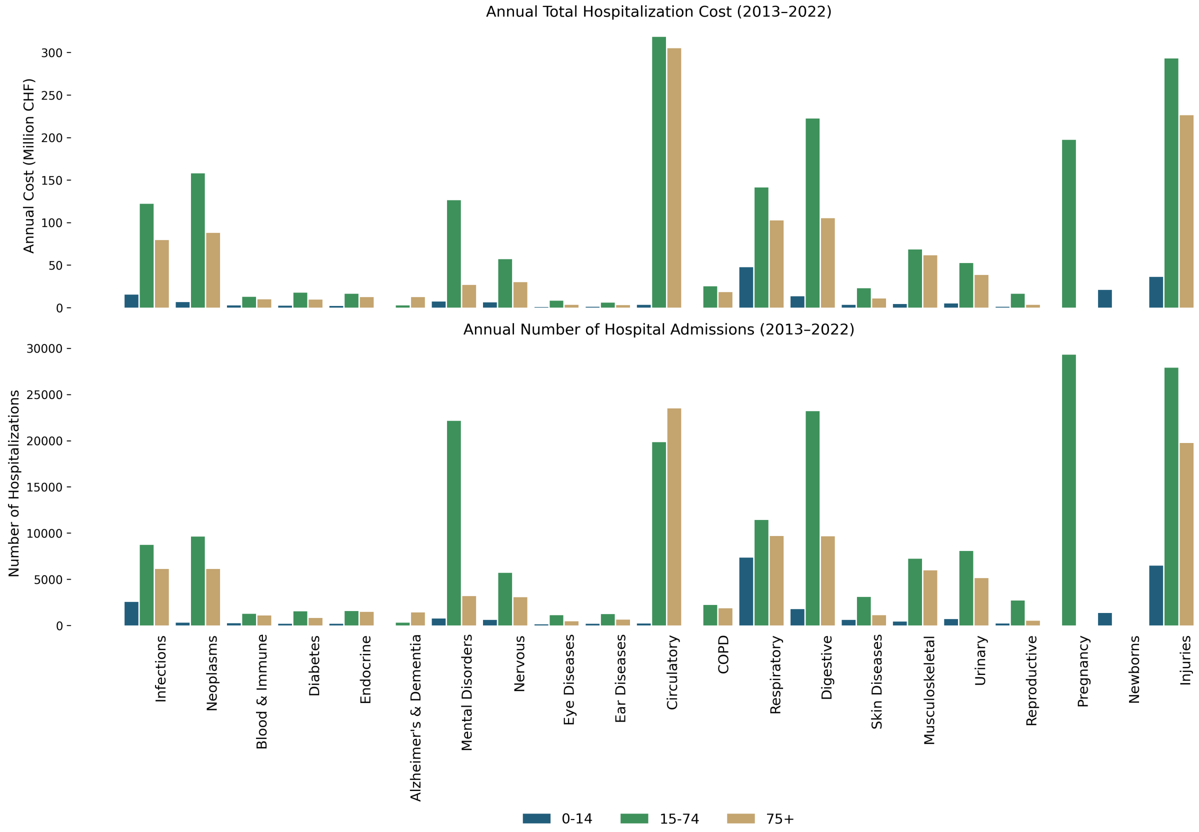

Fig. S6: **Average annual hospitalization costs and number of hospital admissions during the reference period 2013-2022**

Represents the average annual hospitalizations costs for each disease-age group realized between 2013-2022, summing across cantons. The sum of hospital admissions during the whole year across all cantons.

Relative Risk Curves by Canton

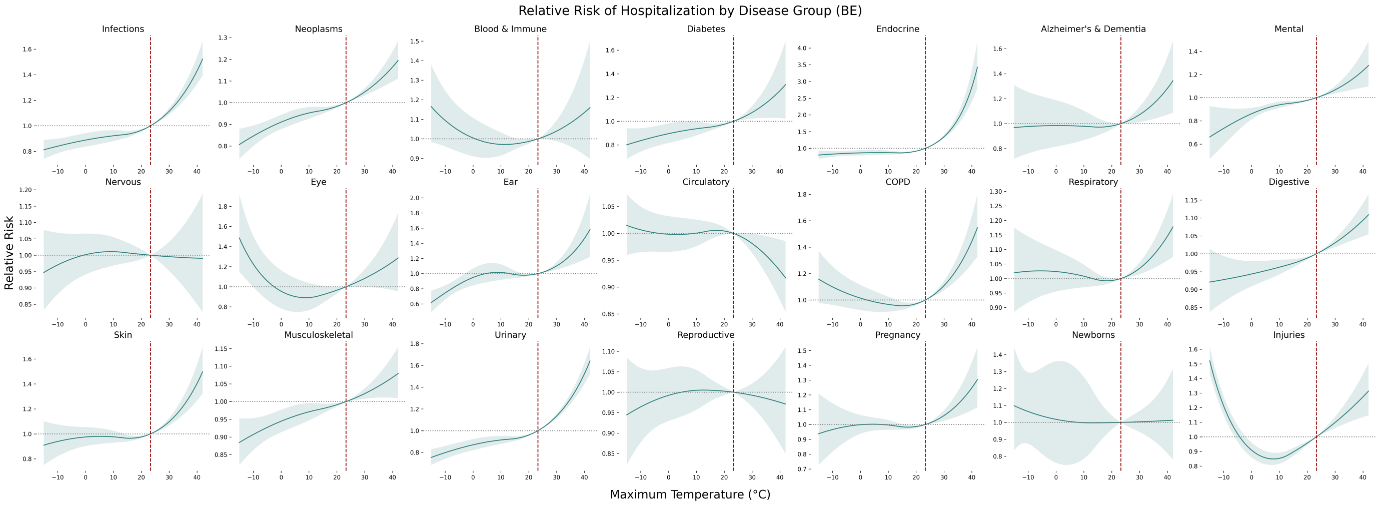

Fig. S7: Bern

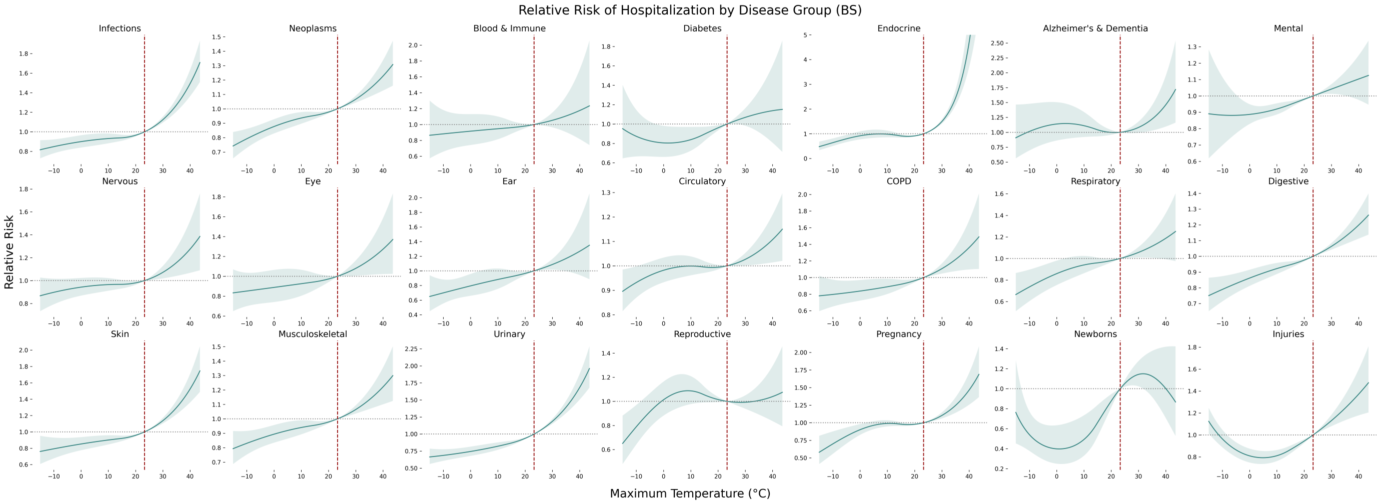

Fig. S8: Basel

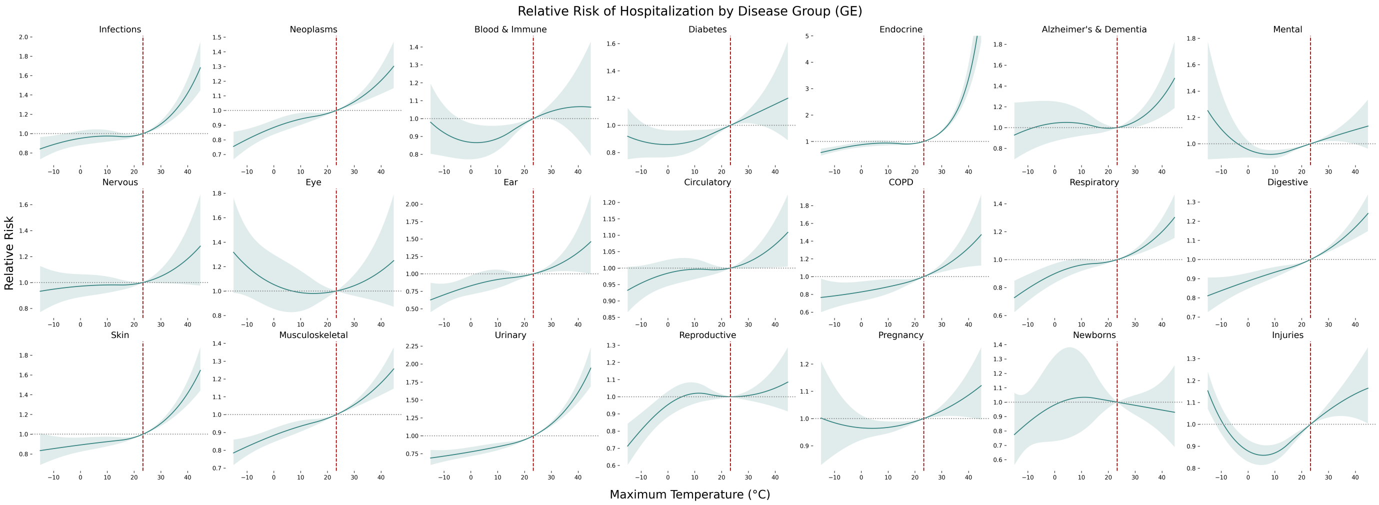

Fig. S9: Geneva

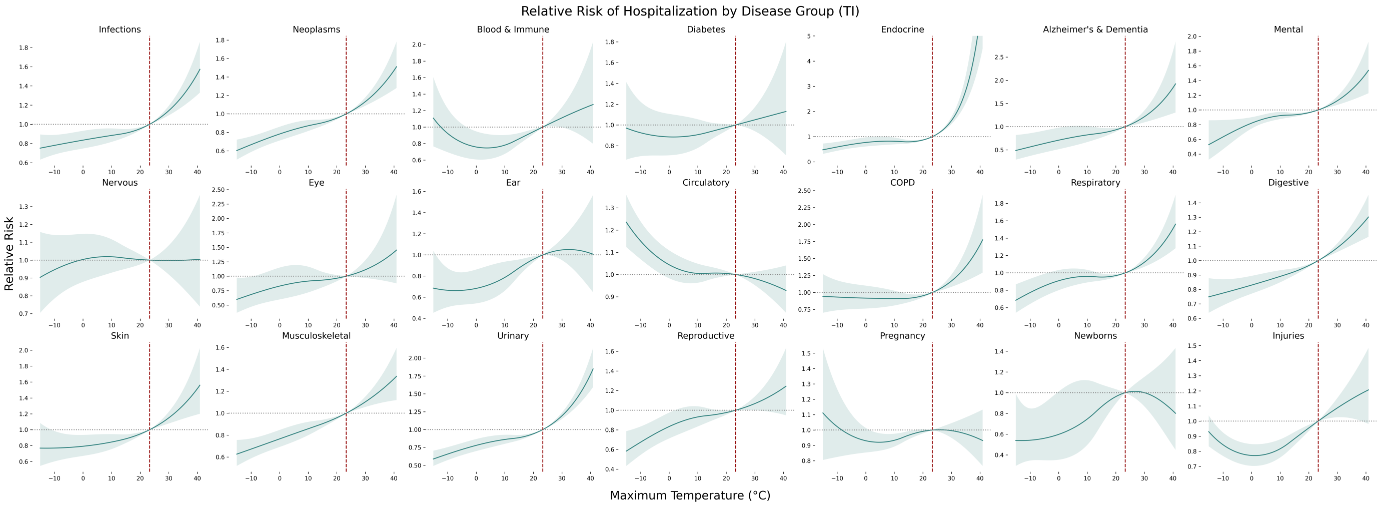

Fig. S10: Ticino

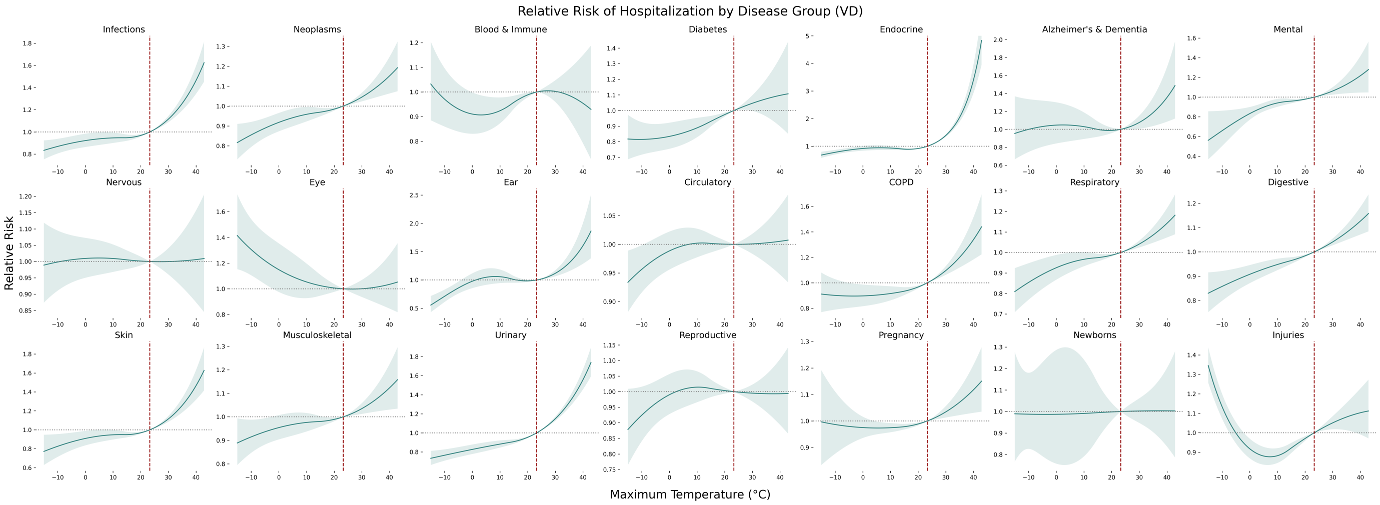

Fig. S11: Vaud

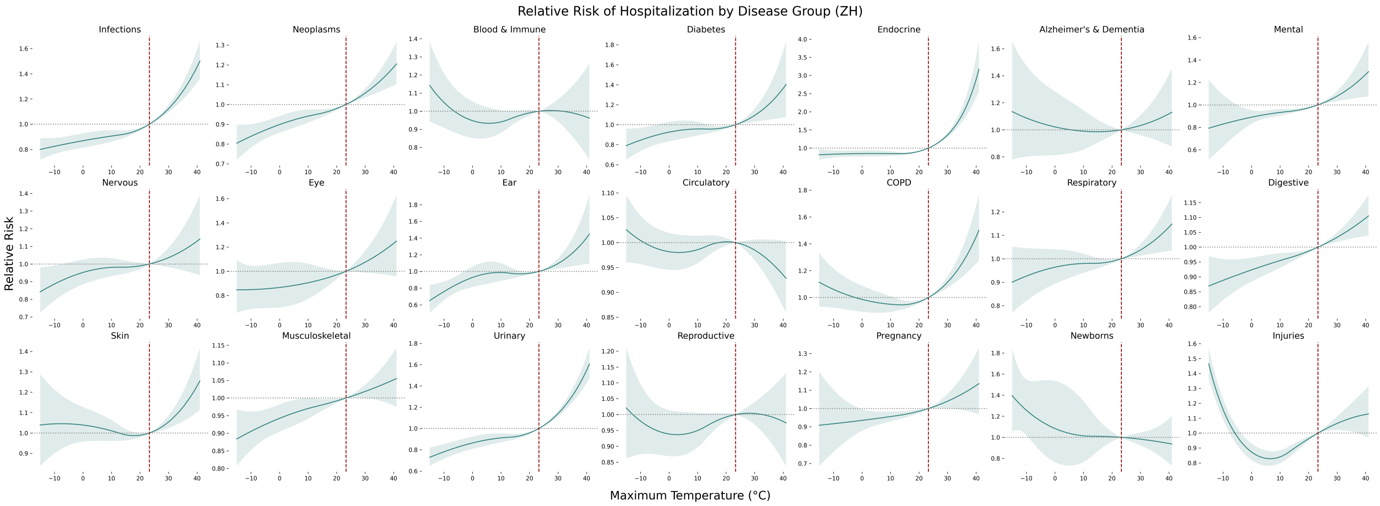

Fig. S12: Zürich

Socioeconomic Influence on Climate Projections: A Comparison of SSP-RCP and RCP-Only Scenarios

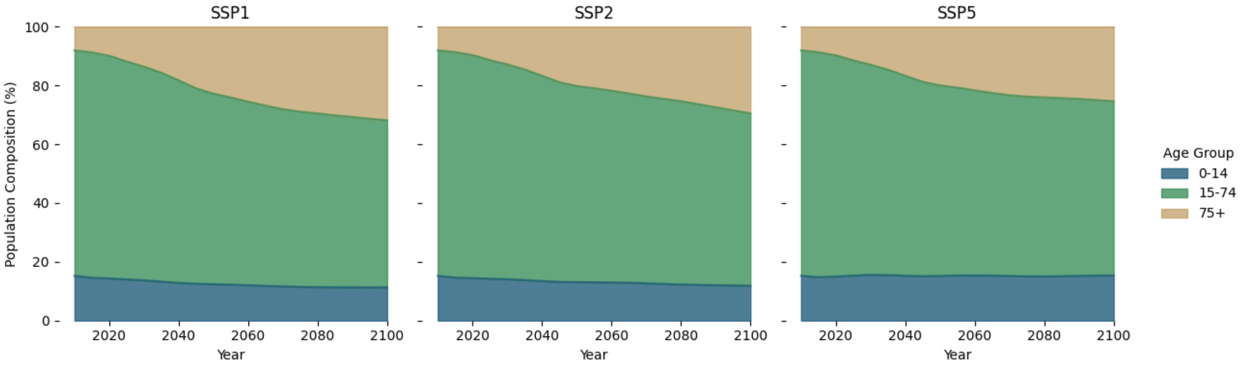

Fig. S13: Demographic Composition under Different SSP Scenarios

Table S1: Summary of Temperature Simulation Models CH2018

lightgray **Institution**	**Regional Model**	**Global Driving Model**	**Resolution**	**Scenario**	**Total Simulations**
DMI	HIRHAM	ECEARTH	EUR11, EUR44	RCP26, RCP45, RCP85	6
KNMI	RACMO	ECEARTH, HADGEM	EUR44	RCP26, RCP45, RCP85	6
MPICSC	REMO1, REMO2	MPIESM	EUR11, EUR44	RCP26, RCP45, RCP85	12
SMHI	RCA	Multiple (ECEARTH, HADGEM, MIROC, etc.)	EUR11, EUR44	RCP26, RCP45, RCP85	30
CLMCOM	CCLM4, CCLM5	Multiple (ECEARTH, HADGEM, etc.)	EUR11, EUR44	RCP45, RCP85	15
ICTP	REGCM	HADGEM	EUR44	RCP85	1

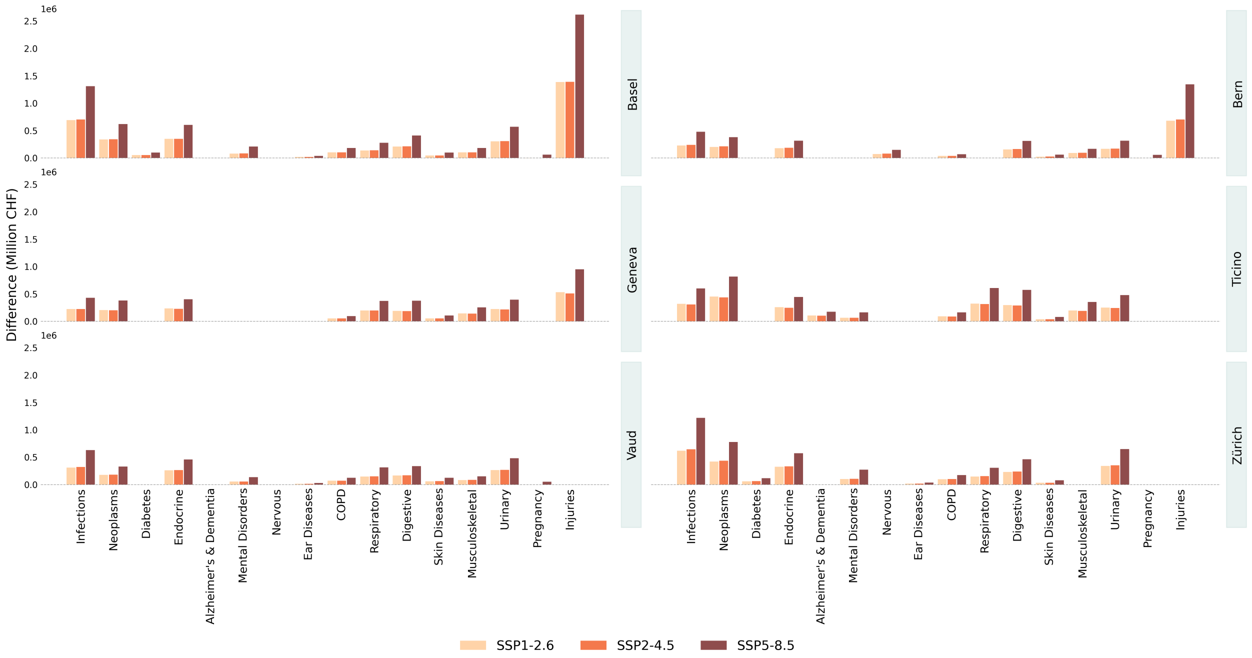

Fig. S14: Difference in results between the cost projections including both SSP and RCP evolution and those 'climate only', assuming no demographic evolution and the same number of hospitalizations as in the reference period 2013-2022. Cost Difference = (Cost increase in SSP-RCP scenarios) - (Cost increase in RCP-only scenarios)

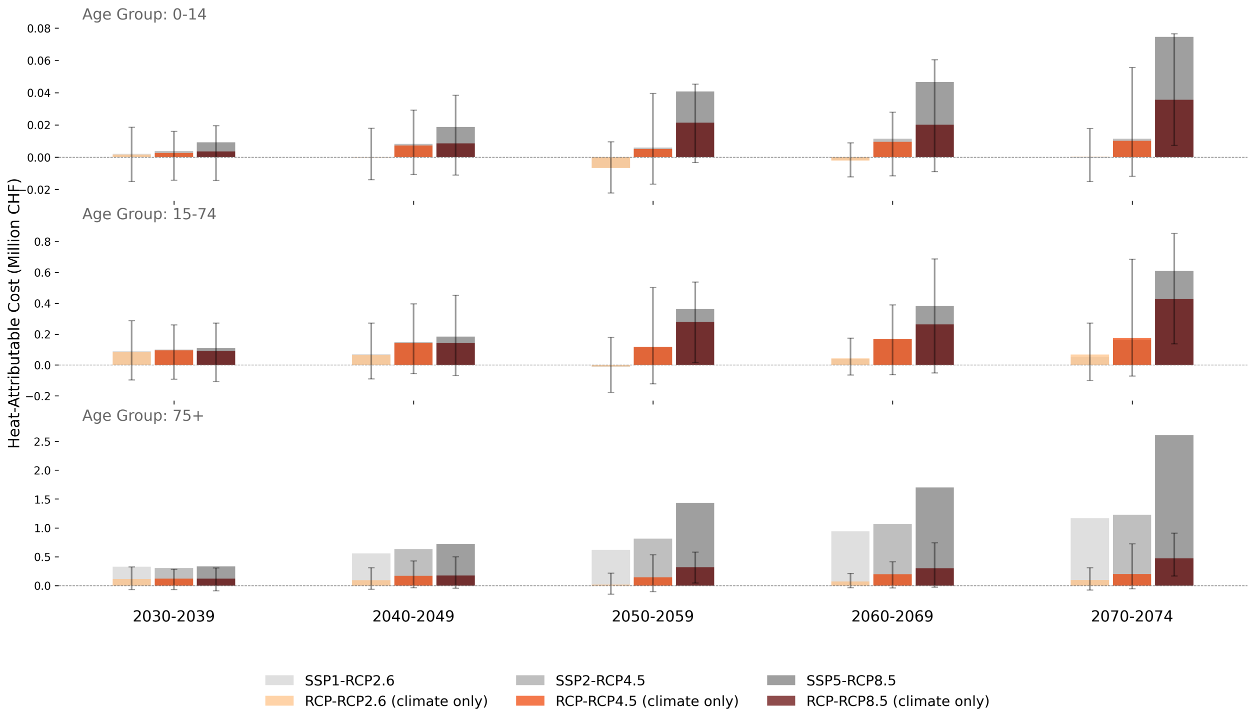

Fig. S15: Difference in results between the cost projections including both SSP and RCP evolution and those 'climate only', assuming no demographic evolution and the same number of hospitalizations as in the reference period 2013-2022. Cost Difference = (Cost increase in SSP-RCP scenarios) - (Cost increase in RCP-only scenarios)

Disease Groups In this study, ICD codes were mapped to 24 mutually exclusive disease categories, primarily based on ICD-10 chapters (letters), with the following modifications.

The genitourinary category (ICD-10 chapter N) was divided into urinary system and reproductive organs (male and female) to isolate the impact on the urinary system, which has been shown in previous research to be affected by heat.

The Newborns category includes ICD-10 codes P00-P96 (Certain conditions originating in the perinatal period) and Q00-Q99 (Congenital malformations, deformations, and chromosomal abnormalities). To maintain consistency with the DRG system and improve interpretability, we excluded codes related to conditions diagnosed at birth but typically treated later in childhood or adulthood (e.g., cleft lip/palate (Q35-Q37), minor limb deformities (Q65-Q79), chromosomal abnormalities (Q90-Q99)). Additionally, since the P00-P96 chapter includes conditions that originate in the perinatal period but may result in morbidity or mortality later, we excluded all patients with an age category above 0-4.

Alzheimer's disease and dementias were separated from the broader category of mental and neurological disorders and grouped into their own category (F00-F03, G30–G31).

Similarly, Diabetes (E10–E14) was extracted as a standalone category, and Chronic Obstructive Pulmonary Disease (COPD) was isolated from the broader respiratory category (J41–J44).

We classified external causes of morbidity (V01–V99, X01–X99, Y01–Y98) under injuries and poisonings, as these codes capture accidents and other external causes of injury. Notably, these codes are only used in the canton of Geneva, whereas in other Swiss cantons, the codes S00–T98 are used instead.

The category Symptoms, signs, and abnormal clinical and laboratory findings, not elsewhere classified (R00–R99) was removed, as these codes represent nonspecific conditions without a definitive diagnosis. Similarly, Factors influencing health status and healthcare use (Z00–Z99) was excluded, as it does not correspond to a disease-based classification.

Finally, special classification codes (U00–U99) were excluded from the analysis, including COVID-19 (U07.1).

For the age-level analysis, we excluded the following disease-age combinations due to a smaller number (¡100) of observations across cantons: Alzheimer's & Dementia in the 0–14 age group, COPD in the 0–14 age group, Pregnancy & Childbirth in the 0–14 and 75+ age groups, and Newborns & Congenitals in the 15–74 and 75+ age groups.

Table S2: Disease Groups

lightgray	**Disease Group**	**ICD-10**	**Swiss DRG**
1	Infectious and parasitic diseases	A00-B99	T
2	Neoplasms	C00-D49	R
3	Blood, hematopoietic organs, and immune system	D50-D89	Q
4	Diabetes	E10-E14	K
5	Endocrine, nutritional, and metabolic diseases without diabetes	E00-E10, E15-E99	K
6	Alzheimer's & Dementia	F00-F03,G30-G31.1,G31.8-G31.9	U, V
7	Mental Disorders excluding Dementia	F04-F99	U, V
8	Nervous system	G00-G99	B
9	Eye and adnexa	H00-H59	C
10	Ear and mastoid process	H60-H95	D
11	Circulatory system	I00-I99	F
12	Chronic Obstructive Pulmonary Diseases (COPD)	J41-J44	E
13	Respiratory system without COPD	J00-J40, J45-J99	E
14	Digestive system	K00-K99	G, H
15	Skin and subcutaneous tissue	L00-L99	J
16	Musculoskeletal system and connective tissue	M00-M99	I
17	Urinary System	N00-N39	L
18	Reproductive Organs	N40-N98	M, N
19	Pregnancy, childbirth and the puerperium	O00-O99	O
20	Newborns & Congenital malformations and deformations	P00-P96, Q00-Q45	P
21	Symptoms & Unclassified	R00-R99	9
22	Injuries, Poisonings & External-Cause Morbidity	S00-S99, T08-T19, T33-T99, V01-V99,X01-X99,Y01-Y99	X, W, Y
23	Other Factors Influencing Health	Z00-Z93	Z
24	Special Classifications (incl. COVID)	U00-U99	-

Sensitivity Analysis Following (1), we selected the number and position of knots based on the Akaike and Bayesian Information Criteria (QAIC and QBIC) fitted through quasi-likelihood:

$$\mathrm{QAIC} = D + 2 \cdot \mathrm{k}, \quad \mathrm{QBIC} = D + \log(n) \cdot \mathrm{k}$$

where D is the quasi-deviance as a proxy for the log-likelihood term $-2\mathcal{L}(\theta)$, k denotes the number of model parameters, and n is the number of observations.

We tested the fit of various model configurations across all combinations of canton and disease groups and selected the specification that minimized each criterion on average. Both criteria selected the model with one internal knot at the 50th percentile as the best model, and the model with one internal knot at the 75th percentile as the second best. Figure 16 shows the relative risk at the 90th percentile of temperature for all 9 model configurations considered, as well as the selected model by setting the maximum lag to 5 and 9 days instead of 7. The results remain qualitatively the same when employing the first or second-best model and, in most cases, also when varying the maximum lag.

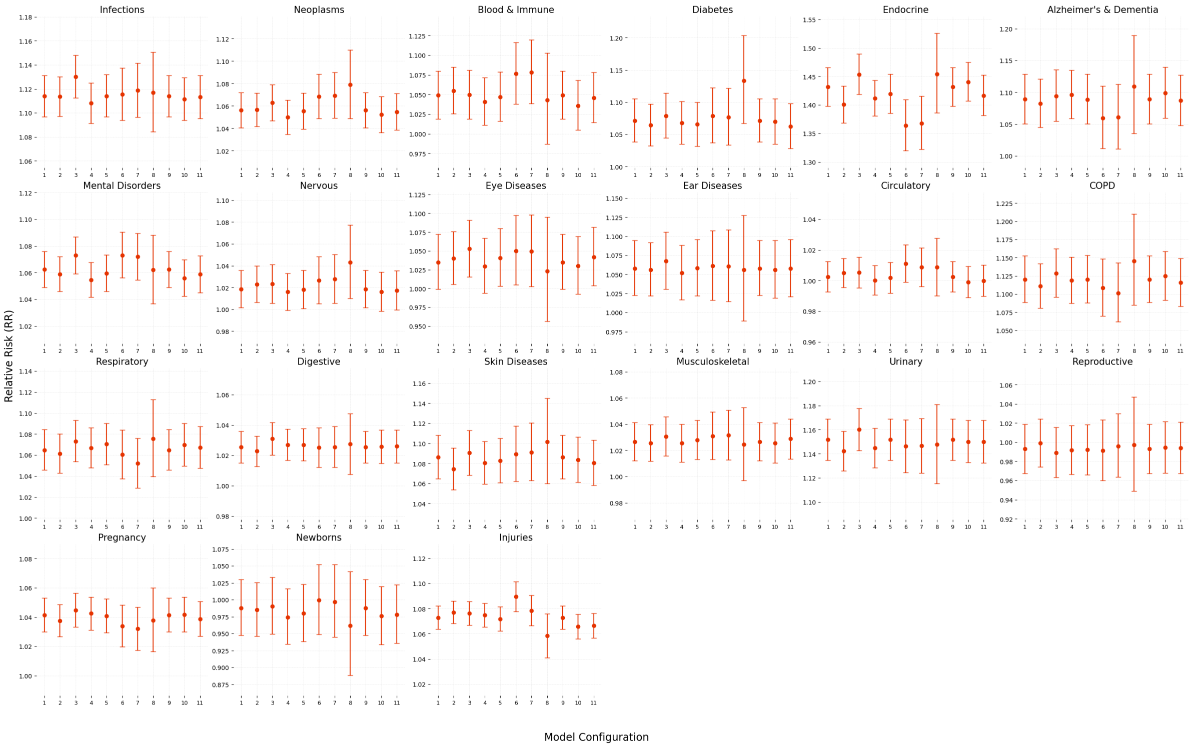

Fig. S16: The 11 model configurations considered are as follows: Model 1 uses a knot at the 50th percentile with MaxLag = 7, Degree = 2, and boundary knots at the minimum and maximum. Models 2 and 3 use the same knot position and spline degree, but with MaxLag = 5 and MaxLag = 9, respectively. Model 4 places a single knot at the 75th percentile, with MaxLag = 7 and boundary knots at the minimum and maximum. Model 5 uses two internal knots at the 50th and 75th percentiles, and Model 6 uses internal knots at the 50th and 90th percentiles. Model 7 includes three internal knots at the 10th, 50th, and 90th percentiles, and Model 8 uses knots at the 25th, 75th, and 90th percentiles. Models 9, 10, and 11 specify splines with 3, 4, and 5 degrees of freedom, respectively, with MaxLag = 7. All models use a quadratic B-spline for the temperature dimension and a natural cubic spline with two internal knots at a logarithmic scale for the lag dimension.

Figure 17 displays the exposure-response curves for different temperature metrics $T_{\min}, T_{\mathrm{mean}}, T_{\max}$.

The curve based on minimum temperature (T_{min}) suggests in most cases a higher risk compared to that based on maximum temperature, while those with T_{mean} lie in between.

It is well established that both high daytime and nighttime temperatures can impact heat-related health outcomes. Although these two measures are usually correlated, high nighttime temperatures may have a distinct effect, especially in older adults, by reducing the body's ability to recover from the heat experienced during the day. In contrast, high daytime temperatures often occur during periods of outdoor activity or work and are more directly linked to heat stress while people are awake and active (2).

Nighttime temperatures are also more influenced by local conditions, such as urban heat islands and the insulation quality of homes. In Switzerland, efforts to reduce heat exposure at night may become more common, such as the development of green areas and the construction of better-insulated buildings. As these measures expand, the relationship between minimum temperature and health outcomes may become more variable and more complex to separate from the effects of such interventions. For this reason, and in line with many existing studies, we used maximum temperature as the primary exposure measure in our analysis. Still, this sensitivity analysis indicates that using the minimum temperature can lead to significantly higher estimates of health risks, and this should be considered when interpreting the results.

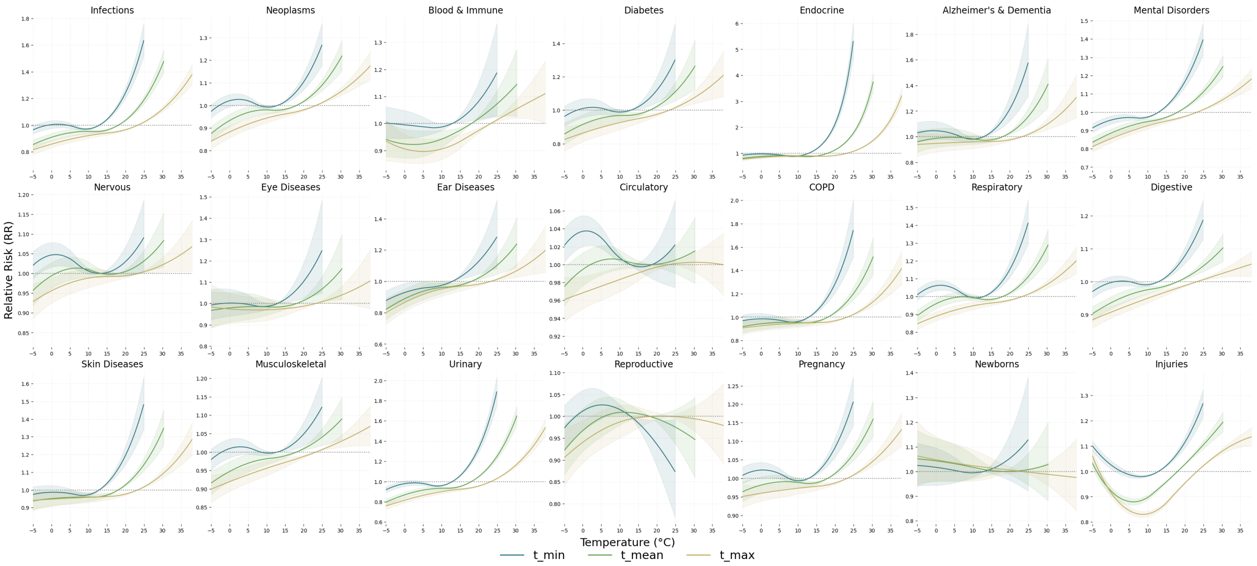

Fig. S17: Comparison of temperature variables

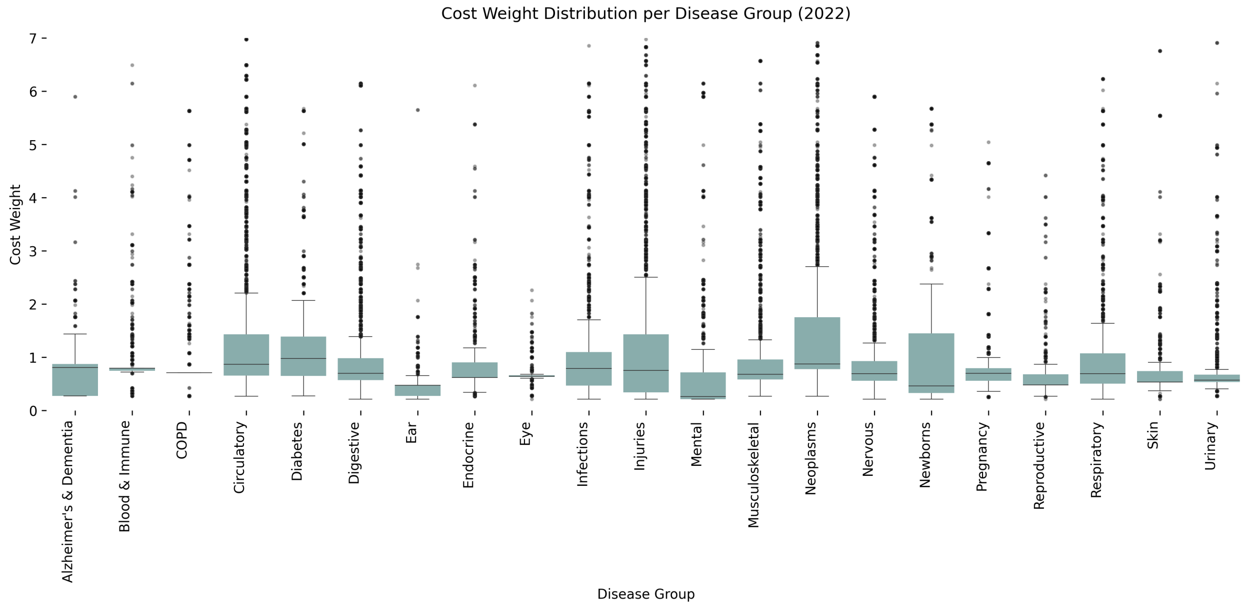

Fig. S18: **Cost Weight Distribution.** Cost weights are calculated for each disease group by averaging the cost weights across all patients with a diagnosis in that group in the year 2022. The cost weights are assigned using the 11th coding version (2022) of the Swiss DRG tariff system.

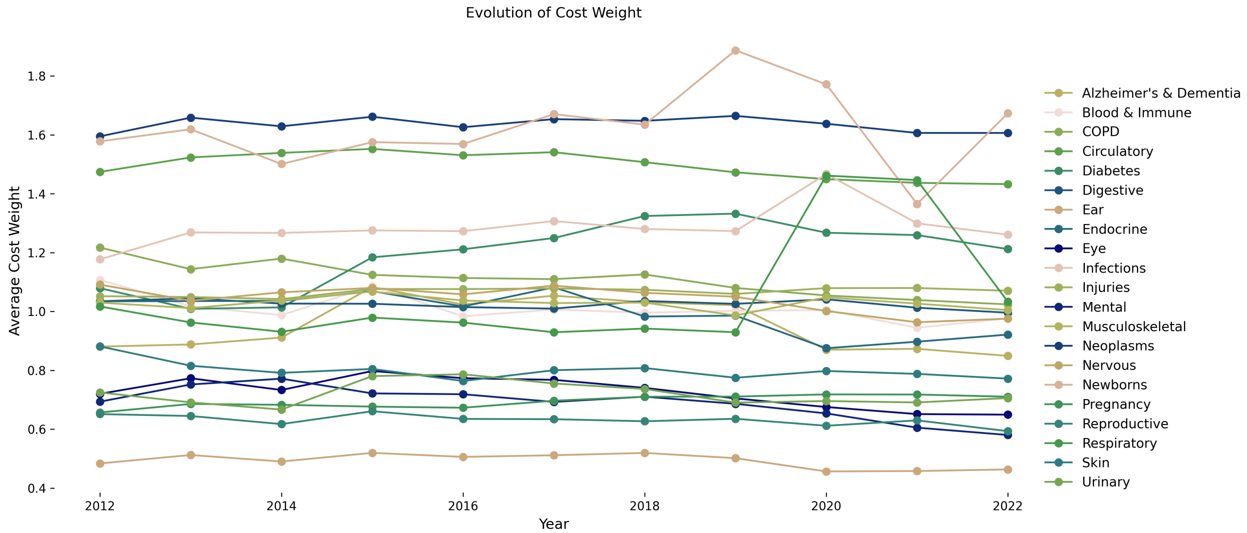

Fig. S19: **Evolution of Cost Weight by Disease Group**

The yearly cost weights are calculated as in (18) for each year from 2012 to 2022. These years correspond to the years when the Swiss DRG information for each patient was available. Each Swiss DRG code was mapped to the corresponding cost using the associated version of the Swiss DRG Tariff, from Version 1.0 (2012) to Version 11.0 (2022).

Results with cantonal median temperature

Table S3: Cantonal temperature statistics for the warm season (May-September).

Percentile (%)	All	ZH	BS	BE	GE	TI	VD
99	34.2	33.4	34.9	33.3	35.5	32.8	34.2
97.5	32.7	32.0	33.4	31.6	34.0	31.9	32.6
95	31.2	30.4	31.9	30.4	32.5	30.9	31.2
92.5	30.3	29.5	30.8	29.7	31.4	30.2	30.4
90	29.7	28.8	30.0	28.9	30.7	29.8	29.4
Median	23.3	22.0	23.3	22.3	24.0	25.1	22.9
Mean	23.2	22.0	23.4	22.2	24.0	24.7	22.8

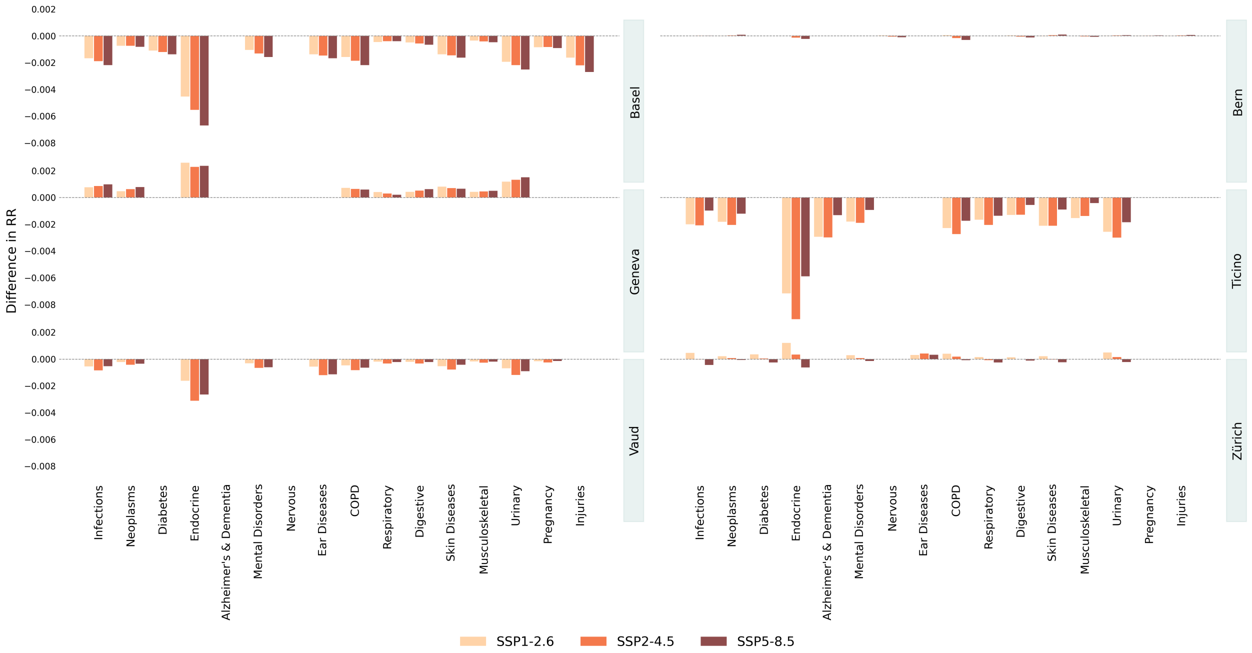

Fig. S20: **Difference between the projected relative risk change using a different reference temperature: Swiss and cantonal warm season's median**
The difference is calculated by subtracting the results obtained using the Swiss warm season's median T_{max} of 23.3 °C as the reference temperature from the results using the cantonal median. Results are the change in relative risk for the period 2013-2022.

Results with disease-specific MMT

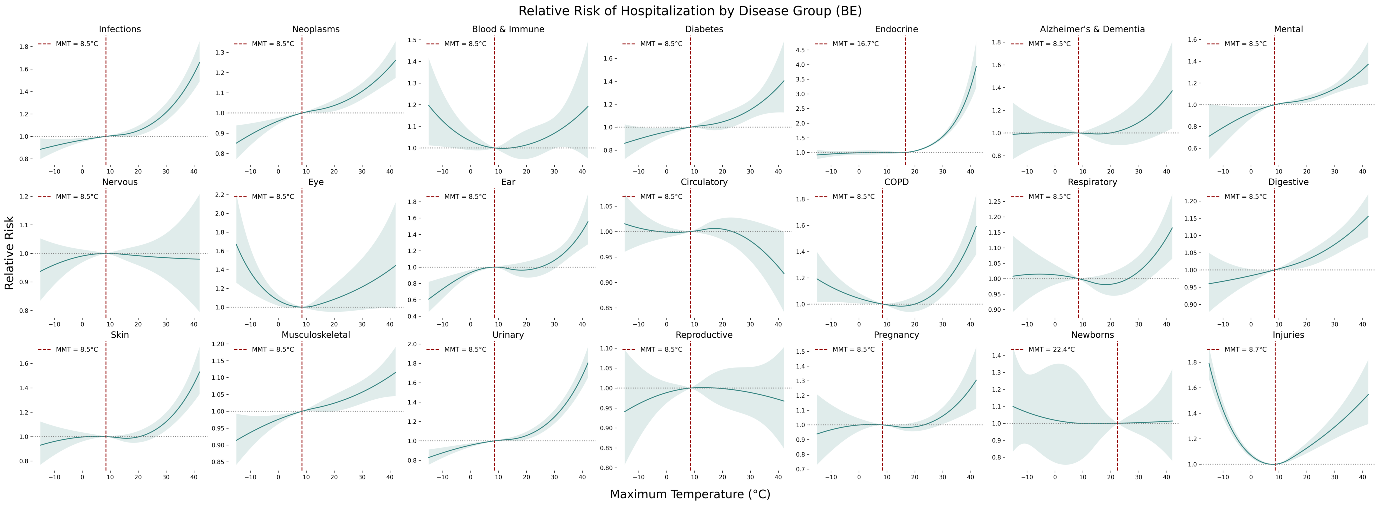

Fig. S21: Bern

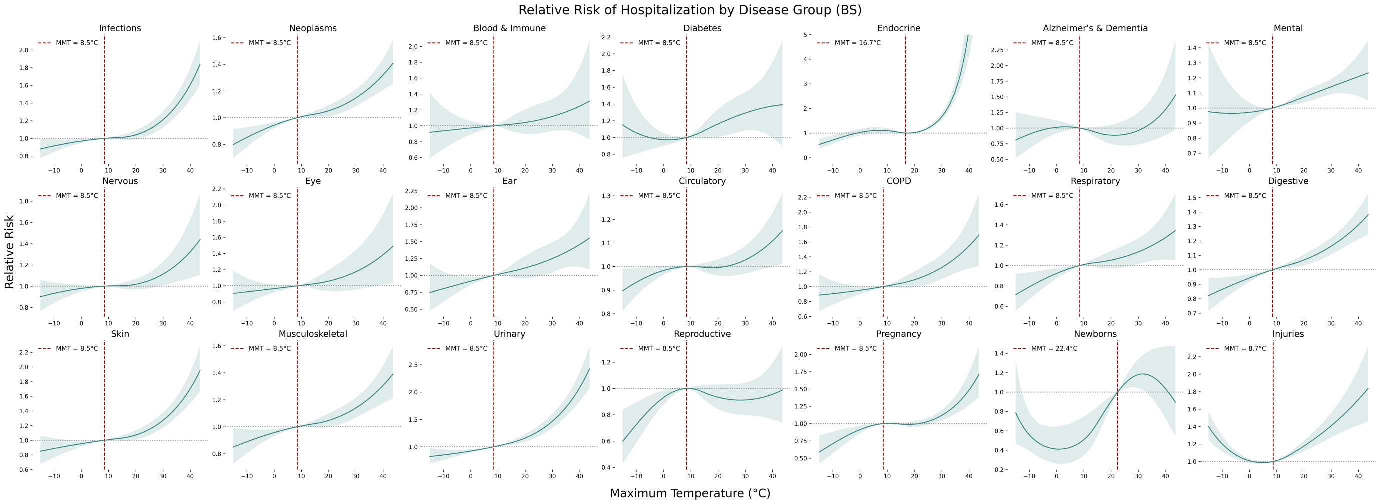

Fig. S22: Basel

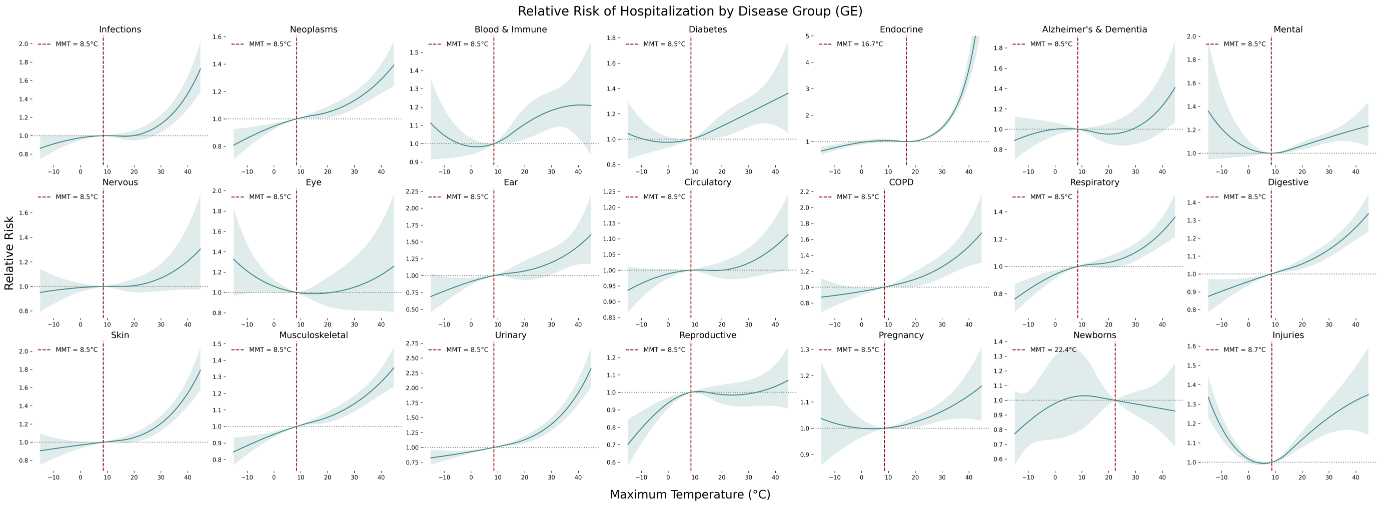

Fig. S23: Geneva

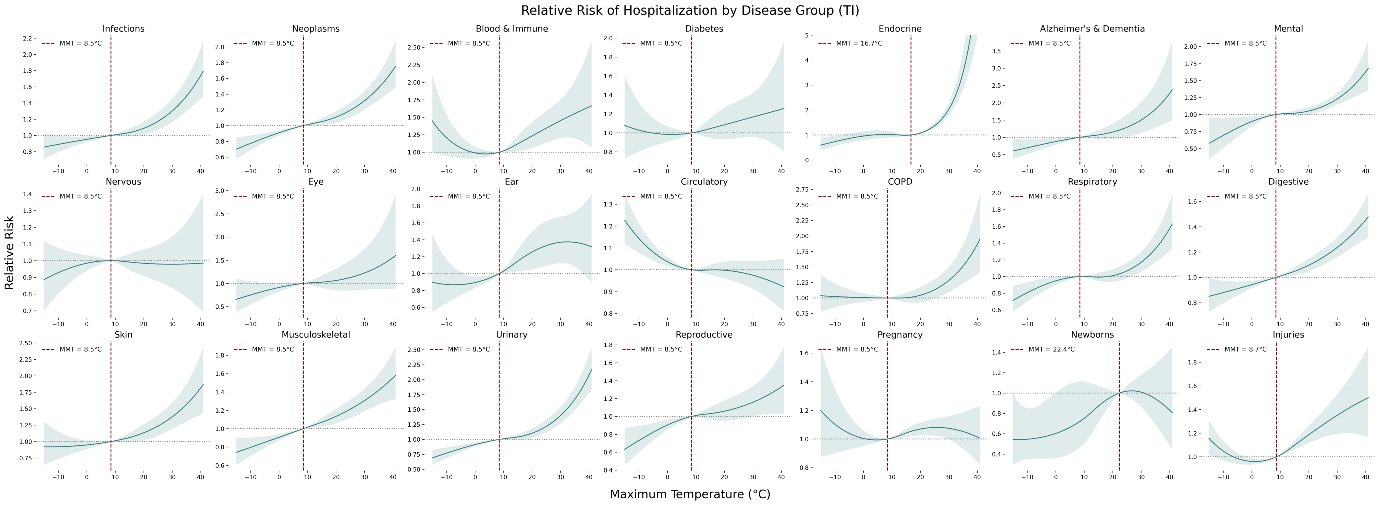

Fig. S24: Ticino

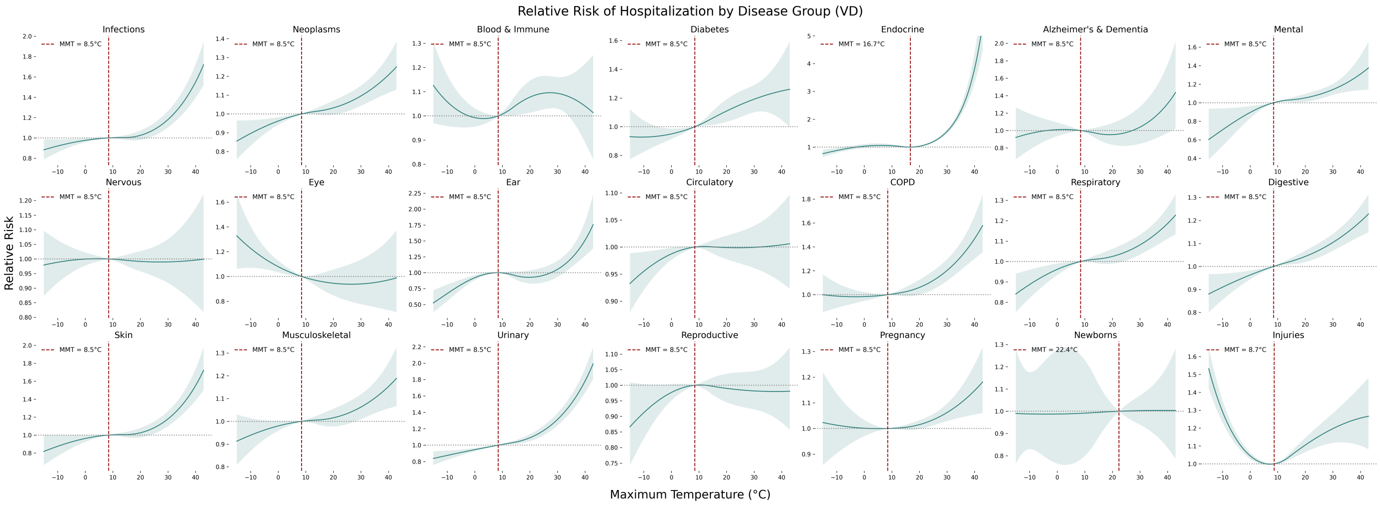

Fig. S25: Vaud

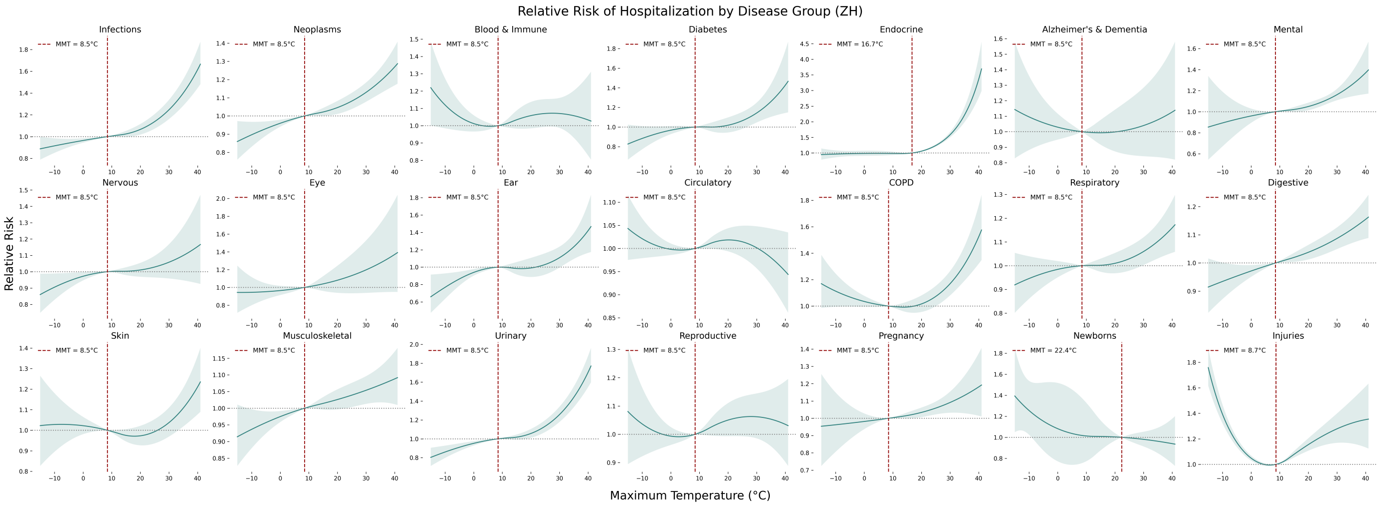

Fig. S26: Zürich

Hospitals base price

Table S4: Hospital-specific base rates as published by individual cantonal authorities. In most cases, multiple base rates from different tariff partners are available for the same hospitals. The displayed value represents the average of these tariffs, which typically show only minor variations.

lightgray **Hospital name**	**canton**	**2012**	**2013**	**2014**	**2015**	**2016**	**2017**	**2018**	**2019**	**2020**	**2021**	**2022**	**2023**	**2024**	**2025**	**2026**
Insel Gruppe AG, Universitätsspital Inselspital	BE				11 000	11 000	11 000	10 800	10 800	10 800	10 800	10 900	10 962	11 107	11 107	
Insel Gruppe AG – Spitäler Aarberg und Riggisberg	BE				9 558	9 685	9 687	9 650	9 655	9 717	9 715	9 715	9 900	10 058	10 026	
Spitalzentrum Biel AG	BE				9 558	9 710	9 686	9 670	9 670	9 717	9 715	9 715	9 900	10 058	10 026	
Spitäler fmi AG	BE				9 558	9 710	9 670	9 652	9 655	9 717	9 715	9 715	9 900	10 058	10 026	
Spital STS AG	BE				9 558	9 685	9 670	9 652	9 655	9 717	9 715	9 715	9 900	10 058	10 026	
Spital Region Oberaargau (SRO) AG	BE				9 558	9 685	9 670	9 652	9 655	9 717	9 715	9 715	9 900	10 058	10 026	
Spital Emmental AG	BE				9 558	9 685	9 670	9 652	9 655	9 717	9 715	9 715	9 900	10 058	10 026	
Hôpital de Moutier SA	BE							9 655	9 717	9 715	9 715			10 059		
Réseau de l'Arc SA	BE				9 558	9 685	9 670	9 652	9 655	9 717	9 715	9 715	9 900	10 058	10 026	
Lindenhofgruppe AG (Engeried, Lindenhof, Sonnenhof)	BE				9 590	9 685	9 670		9 620	9 620	9 687	9 692	9 885	10 026	10 027	
Hirslanden Klinik Linde AG	BE				9 590	9 490	9 608	9 605	9 617	9 593	9 593	9 592	9 592	9 850	9 950	
Siloah AG	BE				9 590	9 490	9 583	9 690	9 600	9 593	9 587	9 580	9 573	9 850	9 850	
Privatklinik Siloah (Swiss Medical Network)	BE					9 578	9 578	9 600	9 600	9 618	9 510	9 510	9 510	9 510	9 510	
Hirslanden Bern AG (Klinik Beau-Site, Klinik Permanence)	BE				9 578	9 578	9 578		9 580	9 577	9 577	9 577	9 898	9 950	9 950	
Klinik Hohmad AG	BE				8 700	8 800	8 797	8 700	8 800	8 900	8 992	9 075	9 075	9 075	9 075	
Geburtshaus Luna AG	BE				9 658	9 658	9 694	9 694	9 694	9 694	9 694	9 694	9 694	9 694	9 694	
Geburtshaus Maternité Alpine	BE					9 694	9 694	9 694	9 694	9 694	9 694	9 694	9 917	10 104	10 104	
Rehaklinik Tschugg AG	BE									9 715	9 600	9 600	9 600			
Hôpital du Jura bernois SA	BE				9 550	9 690	9 670	9 652	9 655	9 717	9 715	9 715				
Spital Basel (USB) Hauptcampus	BS	10 690	10 450	10 430	10 360	10 650	10 650	10 650	10 650	10 650	10 650	10 650	10 650	10 877	11 013	11 150
USB - Universitäre elektive Orthopädie	BS	10 690	10 450	10 430	10 357	10 650	10 650	10 650	10 650	9 630	9 630	9 630	9 630	9 800	9 867	
USB - Augenklinik	BS	10 690	10 450	10 430	10 357	10 650	10 650	10 650	10 650	10 650	10 011	10 011	10 011	10 877	11 013	11 150
Universitäts-Kinderspital Basel	BS	11 367	11 417	11 367	11 350	11 167	10 957	11 000	10 950	10 767	10 767	10 500	10 500	10 500	10 850	10 850
St. Claraspital	BS	9 896	9 756	9 690	9 690	9 677	9 677	9 670	9 670	9 670	9 670	9 670	9 670	9 917	9 947	10 040
Merian Iselin Klinik	BS	9 896	9 896	9 693	9 690	9 635	9 633	9 617	9 608	9 585	9 547	9 572	9 568	9 568	9 817	9 900
Bethesda Spital	BS	9 860	9 771	9 690	9 669	9 667	9 640	9 620	9 600	9 592	9 603	9 575	9 567	9 565	9 800	9 900
Felix Platter Spital	BS	9 900	9 780	9 690	9 644	9 640	9 630	9 593	9 577	9 563	9 563	9 563	9 563	9 782	9 835	
Adullam Spital	BS	9 843	9 682	9 625	9 580	9 567	9 557	9 550	9 550	9 550	9 550	9 550	9 550	9 550		
Schmerzklinik	BS	9 764	9 727	9 680	9 584	9 217	8 930	8 930	8 930	8 930	8 930	8 930	9 205	9 275	9 332	
Palliativzentrum Hildegard	BS									9 453	9 453	9 453	9 453	9 453	9 453	9 453
Geburtshäuser	BS	9 830	9 837	8 819	8 834	8 980	9 100	9 100	9 227	9 210	9 227	9 227	9 227	9 227		
Hôpitaux Universitaires Genève (HUG)	GE							10650	10650	10650	10650	10650	10950	11050		
La Tour Hôpital privé SA	GE							9550	9550	9550	9550	9550	9750	9825		
Clinique de La Plaine (dès 2022)	GE											9550	9375	9750	9825	
Hirslanden Clinique Les Grangettes SA	GE							9550	9550	9550	9550	9550	9750	9825		
Hirslanden Clinique La Colline SA	GE							9550	9550	9550	9550	9550	9750	9825		
Clinique Générale-Beaulieu	GE							9550	9550	9550	9550	9550	9750	9825		
Maison de naissance La Roseraie	GE							9193	9210	9227	9227	9227	9227			
UATM (clinique) d'Onex	GE											5500	6367	6367		
Clinica Ars Medica - Gravesano	TI	9282	9259	9156	9044	9033	8930	8930	8930	8930	8930	8930	9205	9275	9332	
Clinica Sant'Anna - Sorengo	TI	9115	9190	9113	9044	9033	8930	8930	8930	6698	6698	6698	6904	6956	6999	
Clinica Santa Chiara - Locarno	TI	7688	8188	8163	8288	8283	8522	8547	8623							
Ospedale Malcantonese - Castelrotto	TI	6230	6400	6600	6600	6600	6800			8623	8670	8718	8900	8900	9120	9120
Clinica Varini - Orselina	TI	6653	6725	6725	6725	7000	7000	8487	8487	9033	9033	9033	9033	9033		
Ente ospedaliero cantonale EOC - varie sedi	TI	9806	9756	9731	9500	9500	9545	9600	9600	9597	9663	9670	9680	9807	9870	
Clinica Luganese Moncucco SA - Lugano	TI	8275	8200	8125	8280	8280	8500	8500	8500	8700	8700	8700	8900	8900	9120	9120
Fondazione Cardiocentro Ticino - Lugano	TI	9950	9917	9833	9733	9667	9667	9667	9667	9675						
CHUV	VD							10650	10650	9550	10650	10950				
Hôpital ophtalmique Jules-Gonin	VD							10650	10650	10650	10650	10867				
Hôpitaux régionaux membres de la FHV	VD							9600	9650	9650	9663	9908				
Hôpital de Lavaux & Fondation Rive-neuve	VD							9650	9500	9650	9450	9738				
La Source	VD							9500	9500	9450	9500	9750				
Bois-Cerf	VD							9550	9550	9500	9550	9750				
Cécil	VD							9600	9550	9550	9550	9800				
CIC-Riviera	VD							9000	9000	9000	9000	9000				

(continued)

lightgray **Hospital name**	**canton**	**2012**	**2013**	**2014**	**2015**	**2016**	**2017**	**2018**	**2019**	**2020**	**2021**	**2022**	**2023**	**2024**	**2025**	**2026**
Universitätsspital Zürich	ZH	11234	11200	11017	11017	10879	10870	10871	10852	10846	10858	10834	10870	11100	11100	
Kantonsspital Winterthur	ZH	9586	9506	9506	9583	9650	9650	9650	9650	9650	9650	9650	9700	9900	9900	
Stadtspital Triemli	ZH	9500	9500	9500	9670	9700	9700	9700	9784	9776	9838	9900	9940	10067	10200	
Klinik Hirslanden	ZH	9500	9530	9530	9560	9650	9650	9650	9650	9650	9650	9650	9900	9950	9950	
GZO AG Spital Wetzikon	ZH	9586	9506	9506	9583	9650	9650	9650	9650	9810	9850	9850	9900	9950	10050	
Spital Uster	ZH	9586	9506	9506	9583	9650	9650	9650	9650	9810	9850	9850	9900	9950	10050	
Spital Limmattal	ZH	9586	9506	9506	9583	9650	9650	9650	9650	9690	9720	9720	9900	9950	10050	
Spital Bülach	ZH	9586	9506	9506	9583	9650	9650	9650	9650	9810	9850	9850	9900	9950	10050	
Spital Zollikerberg	ZH	9586	9506	9506	9583	9650	9650	9650	9650	9810	9850	9850	9900	9950	10050	
Stadtspital Waid	ZH	9590	9506	9506	9571	9650	9650	9650	9650	9725	9745	9745	9900	9950	10067	
Spital Männedorf	ZH	9586	9506	9506	9583	9650	9650	9650	9650	9810	9850	9850	9900	9950	10050	
Schulthess-Klinik	ZH	9386	9306	9306	9383	9450	9450	9450	9450	9610	9650	9650	9700	9750	9850	
Kinderspital Zürich	ZH	11976	12196	11772	11680	11125	11185	11170	10820	10580	10520	10600	10820	11000	11000	
See-Spital Standort Horgen	ZH	9590	9506	9506	9571	9650	9650	9650	9650	9692	9718	9718	9900	9950	10050	
See-Spital Standort Kilchberg	ZH	9390	9306	9306	9371	9450	9450	9450	9450	9492	9518	9518				
Universitätsklinik Balgrist	ZH	10060	10190	8780	9792	9780	9785	9795	9780	9784	9792	9792	9855	9950	10050	
Spital Affoltern	ZH	9586	9506	9506	9583	9650	9650	9650	9650	9810	9850	9850	9900	9950	10050	
Paracelsus-Spital Richterswil	ZH	9586	9506	9506	9583	9650	9650	9650	9650	9810			9900	9950	10050	
Klinik Lengg	ZH	11498	13816	11326	9948	11528	11485	11556	11634	11360	11200	11200	11200	11200	11200	
Uroviva Klinik für Urologie	ZH	9253	9306	9306	9333	9450	9450	9450	9450	9470	9470	9470	9700	9727	9827	
Uroviva Klinik für Urologie Klinik Susenberg	ZH	9253	9306	9306	9333	9450	9450	9450	9450	9650	9650	9650	9700	9750	9850	
Adus Medica	ZH	9386	9306	9306	9368	9450	9450	9450	9450	9650	9650	9650	9700	9750	9850	
Limmatklinik	ZH	8823	8809	8830	8876	9070	9100	9450	9450	9650	9650	9650	9700	9750	9850	
Geburtshaus Zürcher Oberland	ZH	8823	8809	8830	8876	9070	9100	9100	9160	9192	9212	9236	9300	9227	9227	
Geburtshaus Delphys	ZH							9100	9160	9192	9212	9236	9300	9227	9227	
Geburtshaus Weinland 3	ZH												9227	9227		

Table S5:Projected difference in annual heat-attributable healthcare cost in 2060-206 by disease, age and canton. The reference period is the decade 2013-2022. Cost (in CHF) represent the **difference** between the estimated annual future heat-attributable healthcare costs in the decade 2060-2069 and the historical annual heat-attributable costs registered during the reference period. In brackets the difference in unit of the historical heat-attributable cost is shown, namely: relative cost $= \frac{\text{future cost - cost reference period}}{\text{cost reference period}}$. Only disease-age-canton combination with a significant heat relative risk (relative risk above the heat threshold) are shown.

Disease	Age Group	BE	BS	GE	TI	VD	ZH	Total
**SSP1-2.6**								
Alzheimer's & Dementia	75+	66708 (249%)			109733 (182%)			176441 (202%)
COPD	15-74	2457 (4%)	-5340 (-17%)	-2949 (-9%)	-7164 (-15%)	-3471 (-8%)	-6660 (-9%)	-23130 (-8%)
	75+	92238 (265%)	31761 (188%)	45405 (217%)	79045 (195%)	58889 (221%)	84281 (217%)	391622 (219%)
Circulatory	15-74	-22667 (7%)			27837 (-14%)		25586 (-8%)	30755 (-4%)
	75+		346039 (199%)	446820 (232%)				792859 (216%)
Diabetes	75+	97663 (305%)		34981 (253%)			102364 (252%)	235009 (272%)
Digestive	0-14		-1094 (-7%)	429 (3%)	-605 (-4%)			-1270 (-3%)
	15-74	11019 (7%)	-24501 (-15%)	-11607 (-6%)	-32155 (-13%)	-8909 (-5%)	-15810 (-6%)	-81964 (-7%)
	75+	294219 (279%)	191942 (201%)	237616 (231%)	347901 (209%)	226338 (236%)	316812 (231%)	1614831 (230%)
Ear Diseases	0-14	774 (17%)	-300 (-8%)	66 (2%)	-142 (-5%)	197 (3%)	109 (2%)	705 (3%)
	15-74	104 (1%)				-940 (-11%)		-836 (-5%)
Endocrine	0-14	1476 (36%)	366 (7%)	803 (19%)	533 (10%)	2184 (20%)	1167 (18%)	6531 (18%)
	15-74	-1540 (-2%)	-15248 (-22%)	-8496 (-14%)	-14272 (-20%)	-12680 (-13%)	-16178 (-14%)	-68417 (-14%)
	75+	457798 (276%)	190658 (199%)	261159 (229%)	278120 (207%)	314920 (233%)	412752 (229%)	1915409 (232%)
Infections	0-14	-1993 (-5%)	-11497 (-26%)	-2344 (-18%)	-3880 (-23%)	-8610 (-17%)	-12391 (-18%)	-40719 (-17%)
	15-74	37144 (11%)	-23666 (-13%)	-3812 (-4%)	-19617 (-10%)	-4304 (-2%)	-16178 (-4%)	-30436 (-2%)
	75+	941166 (274%)	282707 (196%)	274193 (226%)	388183 (204%)	400607 (230%)	814625 (226%)	3101484 (232%)
Injuries	15-74	95874 (10%)	-57190 (-12%)	-13965 (-4%)	-39954 (-10%)	-8348 (-2%)	-31338 (-3%)	-54922 (-2%)
	75+	1582335 (294%)	688036 (212%)	560800 (244%)	636435 (221%)	490571 (248%)	1204907 (244%)	5163085 (249%)
Mental Disorders	15-74	-44468 (-21%)			-70324 (-36%)	-36780 (-30%)	-102877 (-31%)	-254450 (-30%)
	75+	338165 (305%)	79186 (220%)	168312 (253%)	189127 (228%)	224033 (257%)	391883 (252%)	1390709 (258%)
Musculoskeletal	15-74		-5233 (-14%)	-1944 (-5%)	-8677 (-11%)			-15854 (-10%)
	75+	287244 (284%)	138493 (204%)	233080 (236%)	267984 (213%)	174638 (240%)	267287 (235%)	1368729 (236%)
Neoplasms	0-14	4423 (17%)	-1698 (-9%)	174 (2%)	-668 (-6%)	122 (2%)	427 (1%)	2781 (2%)
	15-74	9923 (8%)	-16591 (-14%)	-5403 (-5%)	-26896 (-12%)		-11907 (-6%)	-50874 (-7%)
	75+	496258 (287%)	248334 (206%)	265326 (237%)	508841 (215%)	258829 (242%)	591698 (237%)	2369289 (237%)
Nervous	0-14		-2847 (-22%)					-2847 (-22%)
	15-74		-13315 (-17%)					-13315 (-17%)
	75+		70007 (194%)					70007 (194%)
Pregnancy	15-74	21920 (13%)	-23435 (-12%)			-1364 (-1%)		-2880 (-1%)
Respiratory	0-14	-3249 (12%)				457 (-1%)	1255 (-3%)	-1537 (1%)
	15-74	7511 (10%)	-8279 (-13%)	-4056 (-5%)	-15169 (-11%)	-2968 (-3%)	-5402 (-5%)	-28364 (-5%)
	75+	472857 (305%)	188799 (219%)	431324 (252%)	511646 (227%)	421187 (256%)	497127 (251%)	2522943 (252%)
Skin Diseases	0-14	908 (10%)	-1787 (-12%)	-228 (-4%)	-529 (-10%)	-331 (-2%)	-545 (-3%)	-2512 (-4%)
	15-74	2939 (7%)	-6412 (-16%)	-2901 (-7%)	-4704 (-14%)	-3005 (-6%)	-3419 (-8%)	-17504 (-7%)
	75+	61565 (284%)	30364 (202%)	70308 (234%)	49882 (211%)	78371 (238%)		290492 (235%)
Urinary	0-14		-624 (-7%)	132 (2%)	-188 (-5%)			-679 (-4%)
	15-74	14916 (9%)	-14735 (-13%)	-3955 (-5%)	-21021 (-11%)	-4450 (-4%)	-11625 (-5%)	-40871 (-5%)
	75+	466226 (278%)	213327 (200%)	293489 (230%)	334124 (208%)	371399 (234%)	515773 (230%)	2194340 (232%)
**SSP2-4.5**								
Alzheimer's & Dementia	75+	77330 (292%)			118438 (197%)			195769 (225%)
COPD	15-74	22120 (40%)	3891 (13%)	5310 (16%)	4012 (9%)	10327 (23%)	17509 (24%)	63170 (22%)
	75+	106720 (307%)	38125 (225%)	49463 (236%)	86398 (213%)	68506 (257%)	100901 (259%)	450116 (252%)
Circulatory	15-74	-145503 (46%)			-25423 (13%)		-102455 (30%)	-273383 (32%)
	75+		426724 (245%)	501781 (261%)				928506 (253%)
Diabetes	75+	110801 (345%)		37842 (272%)			120863 (297%)	269507 (311%)
Digestive	0-14		5279 (35%)	5481 (43%)	3843 (28%)			14604 (35%)
	15-74	64937 (42%)	26209 (16%)	36384 (20%)	28158 (11%)	45849 (27%)	64699 (26%)	266238 (23%)
	75+	329830 (312%)	227808 (239%)	257415 (250%)	373219 (224%)	258516 (269%)	367180 (268%)	1813971 (258%)
Ear Diseases	0-14	2805 (62%)	1213 (32%)	1177 (38%)	689 (25%)	2827 (45%)	2823 (43%)	11537 (43%)
	15-74	3510 (36%)				1890 (23%)		5400 (30%)
Endocrine	0-14	3628 (90%)	2863 (53%)	2584 (61%)	2386 (46%)	7588 (69%)	4330 (67%)	23382 (64%)
	15-74	28374 (31%)	4626 (7%)	6654 (11%)	1361 (2%)	16436 (17%)	18634 (16%)	76086 (15%)
	75+	511914 (308%)	225049 (234%)	282230 (247%)	294045 (219%)	358522 (265%)	476903 (264%)	2148664 (260%)
Infections	0-14	11486 (31%)	2767 (6%)	1616 (12%)	237 (1%)	8657 (17%)	11064 (16%)	35829 (15%)
	15-74	167314 (47%)	37809 (20%)	28533 (27%)	28284 (15%)	57223 (32%)	132835 (31%)	452000 (31%)
	75+	1069448 (311%)	339747 (235%)	304492 (251%)	419962 (220%)	465376 (267%)	958208 (265%)	3557235 (266%)
Injuries	15-74	420542 (45%)	88161 (19%)	82409 (22%)	54791 (13%)	107226 (28%)	261843 (28%)	1014974 (29%)
	75+	1784372 (331%)	813752 (251%)	605986 (263%)	676909 (235%)	561403 (284%)	1385333 (280%)	5827757 (281%)
Mental Disorders	15-74	11141 (5%)			-34240 (-17%)	-7438 (-7%)	-20481 (-6%)	-51017 (-6%)
	75+	384054 (346%)	94693 (263%)	183833 (276%)	204865 (247%)	259602 (298%)	460228 (296%)	1587276 (294%)
Musculoskeletal	15-74		6993 (19%)	8596 (22%)	9444 (12%)			25034 (17%)
	75+	324977 (322%)	165866 (245%)	254176 (257%)	286801 (228%)	202656 (278%)	313136 (275%)	1547615 (267%)
Neoplasms	0-14	16690 (63%)	6188 (32%)	4436 (38%)	2841 (25%)	2213 (45%)	19254 (43%)	51624 (43%)
	15-74	52532 (45%)	20812 (18%)	22231 (22%)	28476 (13%)		61012 (28%)	185065 (24%)
	75+	561417 (324%)	296616 (246%)	289497 (259%)	547424 (231%)	298153 (279%)	692040 (277%)	2685150 (269%)
Nervous	0-14		1379 (11%)					1379 (11%)
	15-74		10545 (14%)					10545 (14%)
	75+		85926 (238%)					85926 (238%)
Pregnancy	15-74	90534 (53%)	47677 (24%)			59154 (34%)		197366 (36%)
Respiratory	0-14	-14638 (55%)				-17337 (41%)	-18897 (39%)	-50872 (43%)
	15-74	37335 (52%)	12157 (20%)	21672 (24%)	19667 (15%)	27405 (30%)	34280 (30%)	152519 (27%)
	75+	543631 (351%)	227519 (264%)	476573 (279%)	557845 (248%)	488820 (298%)	587651 (297%)	2882042 (288%)
Skin Diseases	0-14	4398 (49%)	3229 (22%)	1632 (26%)	918 (17%)	4827 (33%)	5144 (32%)	20150 (31%)
	15-74	19675 (45%)	6278 (16%)	8206 (21%)	3494 (10%)	13366 (28%)	12965 (30%)	63985 (26%)
	75+	70996 (327%)	36493 (243%)	77448 (258%)	53617 (227%)	91685 (278%)		330241 (268%)
Urinary	0-14		2624 (30%)	2170 (35%)	988 (26%)			5782 (31%)
	15-74	74295 (46%)	19378 (18%)	18202 (22%)	24984 (13%)	36354 (29%)	69348 (30%)	242563 (27%)
	75+	529036 (316%)	252715 (237%)	318585 (250%)	360378 (224%)	427491 (270%)	606378 (270%)	2494586 (264%)

Table S5 (continued)

Disease	Age Group	BE	BS	GE	TI	VD	ZH	Total
**SSP5-8.5**								
Alzheimer's & Dementia	75+	125174 (476%)			213610 (355%)			338785 (389%)
COPD	15-74	54502 (99%)	17199 (56%)	22298 (68%)	29690 (63%)	31577 (71%)	51736 (71%)	207003 (73%)
	75+	171754 (494%)	61354 (361%)	83973 (400%)	155581 (384%)	109389 (411%)	159278 (409%)	741332 (415%)
Circulatory	15-74	-346677 (110%)			-145102 (73%)		-280051 (83%)	-771831 (90%)
	75+		692826 (399%)	862853 (450%)				1555679 (424%)
Diabetes	75+	174696 (544%)		63029 (453%)			187695 (462%)	425421 (492%)
Digestive	0-14		19947 (131%)	20660 (160%)	18737 (135%)			59345 (141%)
	15-74	154276 (99%)	99311 (61%)	134737 (75%)	167471 (66%)	130217 (76%)	179656 (73%)	865669 (74%)
	75+	519376 (491%)	362631 (380%)	432741 (421%)	659071 (396%)	406768 (423%)	569506 (415%)	2950096 (420%)
Ear Diseases	0-14	8082 (180%)	4731 (123%)	4582 (147%)	3528 (127%)	9279 (147%)	9110 (140%)	39312 (145%)
	15-74	9106 (94%)				6209 (74%)		15315 (84%)
Endocrine	0-14	9217 (229%)	8642 (161%)	8029 (189%)	8682 (166%)	20803 (190%)	11629 (181%)	67006 (184%)
	15-74	77795 (84%)	33128 (49%)	37555 (63%)	37570 (52%)	61142 (63%)	68185 (60%)	315378 (63%)
	75+	805026 (485%)	357017 (372%)	474154 (415%)	515620 (384%)	563441 (417%)	739281 (410%)	3454542 (418%)
Infections	0-14	46550 (126%)	35943 (80%)	13652 (103%)	14173 (86%)	51079 (99%)	65403 (94%)	226802 (97%)
	15-74	382264 (108%)	126270 (67%)	93948 (88%)	138533 (72%)	151657 (85%)	344759 (80%)	1237432 (85%)
	75+	1699565 (495%)	545248 (377%)	521107 (431%)	747835 (393%)	740378 (425%)	1497736 (415%)	5751870 (431%)
Injuries	15-74	960751 (102%)	298296 (63%)	282764 (74%)	277904 (66%)	286764 (75%)	689032 (73%)	2795513 (79%)
	75+	2809981 (522%)	1287580 (398%)	1011960 (439%)	1181512 (408%)	880991 (445%)	2133355 (430%)	9305382 (449%)
Mental Disorders	15-74	102996 (47%)			48237 (25%)	37663 (30%)	96512 (29%)	285410 (33%)
	75+	605887 (546%)	150298 (417%)	308122 (462%)	360525 (435%)	409227 (469%)	713156 (459%)	2547217 (472%)
Musculoskeletal	15-74		24568 (66%)	30125 (79%)	51503 (67%)			106198 (70%)
	75+	513858 (508%)	264891 (391%)	428368 (433%)	504612 (401%)	321265 (441%)	487157 (428%)	2520153 (435%)
Neoplasms	0-14	48386 (183%)	24415 (125%)	17389 (150%)	14741 (128%)	7313 (150%)	62591 (141%)	174839 (147%)
	15-74	122844 (105%)	74647 (64%)	78617 (79%)	155939 (69%)		164730 (76%)	596778 (77%)
	75+	887215 (513%)	472744 (393%)	487687 (436%)	965598 (407%)	470470 (439%)	1075272 (431%)	4358988 (436%)
Nervous	0-14		11210 (88%)					11210 (88%)
	15-74		44849 (59%)					44849 (59%)
	75+		139424 (387%)					139424 (387%)
Pregnancy	15-74	203034 (119%)	149585 (74%)			152002 (87%)		504623 (92%)
Respiratory	0-14	-44332 (166%)				-60598 (143%)	-64873 (135%)	-169803 (145%)
	15-74	86063 (120%)	41527 (67%)	73912 (83%)	98804 (74%)	74078 (82%)	90346 (80%)	464732 (83%)
	75+	863887 (557%)	362613 (421%)	804986 (471%)	986008 (438%)	771354 (470%)	913248 (461%)	4702099 (470%)
Skin Diseases	0-14	13656 (153%)	15121 (104%)	7509 (120%)	5963 (109%)	17782 (123%)	18692 (118%)	78726 (121%)
	15-74	47101 (107%)	24511 (62%)	30758 (78%)	22393 (65%)	38412 (80%)	35641 (83%)	198817 (80%)
	75+	113533 (524%)	58422 (389%)	131464 (437%)	94697 (401%)	146074 (443%)		544192 (441%)
Urinary	0-14		10284 (118%)	8534 (136%)	4965 (129%)			23784 (127%)
	15-74	172259 (106%)	68578 (63%)	63655 (78%)	130862 (70%)	99142 (80%)	184199 (79%)	718698 (80%)
	75+	839049 (501%)	401974 (377%)	536552 (420%)	639202 (397%)	675806 (426%)	946367 (421%)	4038951 (427%)

Table S6:Estimated annual heat-attributable healthcare cost in the reference period 2013-2022 by disease, age and canton. Only disease-age-canton combination with a significant heat relative risk (relative risk above the heat threshold) are shown.

Disease	Age Group	BE	BS	GE	TI	VD	ZH	Total
Alzheimer's & Dementia	75+	26874			60262			87136
Circulatory	15-74	-317256			-200792		-340475	-858523
	75+		174096	193014				367110
COPD	15-74	55289	30681	32461	46941	44172	72518	282063
	75+	34783	16901	20899	40501	26596	38916	178597
Diabetes	75+	32047		13847			40633	86527
Digestive	0-14		15248	12994	13863			42105
	15-74	155432	163093	179380	252342	171183	243823	1165253
	75+	105448	95447	102771	166294	95996	136903	702860
Ear Diseases	0-14	4502	3854	3136	2780	6325	6507	27104
	15-74	9656				8486		18142
Endocrine	0-14	4086	5395	4270	5251	11015	6444	36460
	15-74	92121	68112	59976	71658	96311	113402	501580
	75+	166000	96043	114197	134202	135287	180435	826164
Infections	0-14	37070	44938	13364	16594	51624	69536	233126
	15-74	353563	188087	107878	191749	179518	430462	1451258
	75+	343729	144586	121211	190566	174107	361084	1335282
Injuries	15-74	942836	471484	380323	414487	380074	939874	3529078
	75+	538617	323857	230218	287835	197552	493929	2072008
Mental Disorders	15-74	211716			196071	121637	330207	859632
	75+	111038	36055	66597	82927	87164	155485	539265
Musculoskeletal	15-74		37197	38039	76187			151423
	75+	100953	67774	98963	125640	72811	113614	579756
Neoplasms	0-14	26636	19665	11694	11552	4920	44539	119006
	15-74	116996	116356	99459	226011		214736	773557
	75+	173054	120413	111739	237084	107011	249457	998759
Nervous	0-14		12753					12753
	15-74		76145					76145
	75+		36067					36067
Pregnancy	15-74	171187	203041			172853		547081
Reproductive	75+	4286			18346	4981	7345	34959
Respiratory	0-14	-26595				-42717	-48049	-117361
	15-74	73493	61608	88956	133331	90474	112323	560186
	75+	155105	86225	171115	225425	164211	198034	1000115
Skin Diseases	0-14	8920	14594	6234	5440	14438	15597	65223
	15-74	44009	39701	39558	34348	48021	43212	248849
	75+	21704	15017	30082	23615	32973		123391
Urinary	0-14		8719	6175	3811			18705
	15-74	162144	109198	81896	186117	123919	233943	897218
	75+	167466	106735	127637	160921	158560	224686	946005
Total		4106907	3009085	2568085	3441362	2739502	4689121	20554061

References

[1] Gasparrini A, Armstrong B, Kenward MG. Distributed lag non-linear models. Statistics in Medicine. 2010;29(21):2224-34.

[2] Ragettli MS, Saucy A, Flückiger B, Vienneau D, De Hoogh K, Vicedo-Cabrera AM, et al. Explorative Assessment of the Temperature–Mortality Association to Support Health-Based Heat-Warning Thresholds: A National Case-Crossover Study in Switzerland. International Journal of Environmental Research and Public Health. 2023;20(6):4958. Available from: `https://doi.org/10.3390/ijerph20064958`.
